# Interactions of Opioids and HIV Infection in the Pathogenesis of Chronic Pain

**DOI:** 10.3389/fmicb.2016.00103

**Published:** 2016-02-10

**Authors:** Bolong Liu, Xin Liu, Shao-Jun Tang

**Affiliations:** ^1^Department of Neuroscience and Cell Biology, University of Texas Medical Branch, GalvestonTX, USA; ^2^Department of Urology, Third Affiliated Hospital of Sun Yat-Sen UniversityGuangzhou, China

**Keywords:** HIV-1, opioids, gp120, morphine, neuropathic pain, glia, neuron

## Abstract

Over 50% of HIV-1/AIDS patients suffer chronic pain. Currently, opioids are the cornerstone medications for treating severe pain in these patients. Ironically, emerging clinical data indicates that repeated use of opiate pain medicines might in fact heighten the chronic pain states in HIV patients. Both laboratory-based and clinical studies strongly suggest that opioids exacerbate the detrimental effects of HIV-1 infection on the nervous system, both on neurons and glia. The combination of opioids and HIV-1infection may promote the damage of neurons, including those in the pain sensory and transmission pathway, by activating both caspase-dependent and caspase-independent pro-apoptotic pathways. In addition, the opiate-HIV-1 interaction may also cause widespread disturbance of glial function and elicit glial-derived pro-inflammatory responses that dysregulate neuronal function. The deregulation of neuron-glia cross-talk that occurs with the combination of HIV-1 and opioids appears to play an important role in the development of the pathological pain state. In this article, we wish to provide an overview of the potential molecular and cellular mechanisms by which opioids may interact with HIV-1 to cause neurological problems, especially in the context of HIV-associated pathological pain. Elucidating the underlying mechanisms will help researchers and clinicians to understand how chronic use of opioids for analgesia enhances HIV-associated pain. It will also assist in optimizing therapeutic approaches to prevent or minimize this significant side effect of opiate analgesics in pain management for HIV patients.

## Introduction

Pathological pain is a major neurological complication suffered by over 50% of HIV-1/AIDS patients (Hewitt et al., 1997; Mirsattari et al., 1999; Evers et al., 2000). Patients with HIV-associated pain syndromes may suffer headache, somatic pain, and visceral pain (Hewitt et al., 1997; Mirsattari et al., 1999; Evers et al., 2000; Aouizerat et al., 2010). The pain is typically bilateral, of gradual onset and described as ‘aching,’ ‘painful numbness,’ or ‘burnings’ (Cornblath and McArthur, 1988). However, the molecular and cellular processes by which HIV patients develop pain remain elusive. Pathologically, about 30% of HIV-1/AIDS patients with pain symptoms manifest clinically detectable peripheral neuropathy (Martin et al., 2003). Although highly active antiretroviral therapy (HAART) has dramatically reduced the morbidity and mortality of HIV-1 infection (Mocroft et al., 2003), patients on HAART still develop symptoms of distally symmetric small-fiber retrograde axonal neuropathy and pain (Simpson, 2002; Luciano et al., 2003; Lopez et al., 2004; Arenas-Pinto et al., 2008). Previous studies indicate that gp120 can induce cutaneous denervation in the transgenic mouse model (Keswani et al., 2006), intrathecal injection model (Milligan et al., 2001; Yuan et al., 2014), and in the sciatic nerve exposure model (Wallace et al., 2007a,b). Gp120 can also cause axonal injury of sensory neurons in culture (Melli et al., 2006; Hoke et al., 2009).

Controlling pain is a big challenge in HIV patient care. The clinical practice for pain management in HIV patients has been recommended to follow the WHO guideline (World Health Organization, 1996; Basu et al., 2007). According to the guideline, opioids are the cornerstone medications for treating moderate to severe pain. Ironically, besides the powerful acute analgesic effect, emerging clinical data indicate that repeated use of opioid analgesics promotes chronic pain in HIV patients (Smith, 2011; Onen et al., 2012). Studies on simian or simian-human immunodeficiency virus-infected monkey models suggest a role of opioids in HIV-related disease progression (Kumar et al., 2004, 2006; Rivera-Amill et al., 2010). HIV-1-infected opioid abusers also appear to show more severe neuropathology than HIV-1-infected non-drug users (Bell et al., 1998, 2006; Anthony et al., 2005, 2008). Currently, the mechanisms by which opioid medicines exacerbate HIV- associated pain are unclear. Multiple molecular systems in neurons, including mu-opioid receptors, *N*-methyl-D-aspartate receptors, nitric oxide synthase, heme oxygenase, 5-hydroxytryptamine type 3 receptors, complement components, chemokines and the melanocortin system have been implicated in opiate-induced hyperalgesia (Basu et al., 2007), but whether (and how) they contribute to the exacerbation of HIV-associated pain is unclear. Opioids can also activate glial cells such as microglia and astrocytes (Tortorici et al., 1999; Song and Zhao, 2001; Raghavendra et al., 2002; Mika, 2008; Horvath and DeLeo, 2009; Mika et al., 2009; Lee et al., 2011), which may facilitate the expression of hyperalgesia by releasing various neural regulators such as cytokines, chemokines and brain-derived neurotrophic factor to induce sensitization of pain-processing neurons (Hao, 2013).

Although we have a significant understanding of the potential mechanisms by which HIV-1 infection (including antiretroviral therapy) and opioid use induce pain individually, little is known about how their interaction would contribute to pain pathogenesis. We will consider the potential pathogenic mechanisms from the perspective of neuron-glial crosstalk. In the following sections, we will first provide overviews of the detrimental effects of HIV-1 and opioids, separately or combinatorially, on neurons and glia. Based on these findings, we will discuss the possible pathogenic processes induced by HIV-1 and opioids that facilitate the development of HIV-associated pain. We regret that due to limited space we cannot cover all of the significant work in this field but are forced to focus on selected findings to illustrate our views. The purpose of this paper is not to provide a systematic review of current literature. Instead, we aim to provide mechanistic viewpoints on how HIV-1 infection might interact with opioids to promote pain pathologies.

## Neuronal Mechanisms

We postulate that neuronal damage is a major mechanism by which interaction of HIV-1 and opioids facilitates the development of hyperalgesia. Here, we will first discuss how nerve damage can lead to the expression of pathological pain and then how gp120 and opioids may cause neuronal damage.

Damage of peripheral or central pain transmission neurons, manifested by hyper-excitability and/or a lowered threshold of activation, directly contributes to neuropathic pain (Baron, 2000; Treede et al., 2008). Pathological pain in HIV patients is frequently associated with peripheral sensory neuropathy, a form of the so-called ‘dying-back’ degeneration of sensory neurons (Hao, 2013). Sensory neuropathy also develops in various animal models of HIV-associated pain, including rodents models generated by exposure of peripheral nerves or spinal cord to gp120 (Herzberg and Sagen, 2001), gp120 transgenic mice receiving antiretroviral drugs (Keswani et al., 2006) and SIV-infected monkeys (Hou et al., 2011). Thus, neuronal damage is intimately associated with the expression of HIV-associated pain. Many events such as neuronal hyper-excitation, inflammation and viral infection can cause nerve damage that leads to neuropathic pain (Woolf and Mannion, 1999). Neuropathic pain-related damage may lead to neuronal apoptosis via caspase-dependent and -independent pathways (Perl and Banki, 2000; Oh et al., 2001; Gougeon, 2003; Silva et al., 2003). Using HIV-1 gp120 protein as an example, we will describe how HIV-1 infection may interact with opioids to promote these pathways in pain-processing neurons.

Damage of peripheral nerves may contribute to pain pathogenesis via various pathways. For instance, when peripheral nervesare damaged, sodium-channels may aggregate locally and/or in cell bodies, which may lead to hyper excitability (Lai et al., 2003; Wood et al., 2004). Ectopic expression of specific calcium channels on DRG cells has also been observed following neuronal damage (Luo et al., 2001). The nerve-damage-induced changes of ion channel expression may be intimately linked to peripheral sensitization. In addition to ion channels, neuronal damage induces the ectopic expression of specific pain sensory receptors such as TRPV1, a vanilloid receptor for thermal sensation. TRPV1 is normally expressed on nociceptive afferent fibers. When nerve damage occurs, TRPV1 expression decreases on injured afferents and increases on undamaged Cfibers and Aδfibers (Hudson et al., 2001; Hong and Wiley, 2005). Nerve-damage-induced increases of other pain-related factors, including acid-sensing ion channels (ASICs; Price et al., 2001), adrenoceptors in neurons (Price et al., 1998; Baron et al., 1999) and pro-inflammatory cytokines (e.g., TNF-α; Marchand et al., 2005) in glial cells, have also been implicated in peripheral sensitization.

The sensitization of primary afferent nerves can facilitate the expression of central sensitization of CNS pain-processing neurons in the spinal cord dorsal horn and supraspinal regions (Price, 2000; Zhuo, 2002). Peripheral nerve damage can lead to the activation of excitatory glutamate receptors such as NMDARs and AMPARs in spinal neurons (Miller et al., 2011). In addition, damage to peripheral nerves also causes reduced expression and uptake activity of both neuronal and glial glutamate transporters, which may contribute to increased neuronal excitability (Sung et al., 2003); these effects are mediated by the activation of PKC and MAPK signaling pathways (Malmberg et al., 1997; Ji and Woolf, 2001). Furthermore, hyperactivation of Cfibers induces ectopic expression of sodium channels (Hains et al., 2004) and calcium channels (Luo et al., 2001) on dorsal horn neurons to facilitate pain transmission. Malfunction of inhibitory mechanisms may also facilitate central sensitization. Peripheral nerve damage can induce the apoptosis of GABA (γ-aminobutyricacid) inhibitory neurons in superficial layers of the dorsal horn (Moore et al., 2002; Coull et al., 2003). Several studies suggest an association of neuronal apoptosis with neuropathic pain (Mao et al., 2002a; Moore et al., 2002; Campana and Myers, 2003; Schmeichel et al., 2003), and inhibition of apoptosis decreases the pain behaviors (Joseph and Levine, 2004; Scholz et al., 2005; Sekiguchi et al., 2009).

HIV-1 does not infect neurons (Lipton, 1998; Michaels et al., 1988). However, HIV-1 infection of the nervous system, especially of microglia and astrocytes, can cause neuronal damage and apoptosis via toxic viral proteins that are secreted from infected cells. Glycoprotein 120 (gp120), the viral envelope protein that mediates HIV infection, is one of the secreted HIV-1 proteins that causes neuronal dysfunction (Michaels et al., 1988; Nath, 2002). Our recent analysis on HIV patient tissues and mouse models suggests a crucial role of gp120 in the pathogenesis of HIV-associated pain (Yuan et al., 2014). Gp120 may induce neurotoxicity either by directly stimulating neurons (“direct injury”) or indirectly by activating glial cells (“bystander effect”; Kaul et al., 2001). For instance, gp120 activates C-X-C chemokine receptor 4 (CXCR4), which is constitutively expressed on DRG and spinal cord neurons (Oh et al., 2001; Miller et al., 2009), and up-regulates C-C chemokine receptor 2 (CCR2) in a calcium-dependent manner to produce neurotoxicity (Hesselgesser et al., 1997; Jung and Miller, 2008). It has been suggested that gp120-induced neuronal CXCR4 activation may up-regulate pro-inflammatory cytokine IL-1β in a neuronal autocrine fashion, which then causes the neuronal excitotoxicity (Bagetta et al., 1999; Corasaniti et al., 2001a,b). In addition, gp120 is known to stimulate CXCR4 on DRG satellite glia and induce the secretion of RANTES (Regulated on Activation, Normal T cell Expressed and Secreted) chemokine (a.k.a. CCL5), which induces neuronal damage by activating CCR5 receptors on DRG neurons (Hesselgesser et al., 1997; Oh et al., 2001). Glutamate receptors (Mattson et al., 2005), especially NMDA receptors (Lipton et al., 1991; Lipton, 1992; Bennett et al., 1995; Lannuzel et al., 1995; Meucci and Miller, 1996; Chen et al., 2005), are targets of gp120, and their over-activation by gp120 can cause neurotoxicity.

There are three apoptotic pathways: caspase-dependent extrinsic (also known as death receptor approach) and intrinsic (known as mitochondrial approach) pathways (Sinkovics, 1991) as well as a caspase-independent pathway that is T cell-mediated and exhibits perforin-granzyme-dependent apoptosis (Elmore, 2007). Gp120 can induce neuronal apoptosis via the extrinsic and intrinsic pathways (Bagetta et al., 1999; Chen et al., 2005, 2011a; Singh et al., 2005). In the extrinsic pathway, TNF-α secreted by gp120-activated glial cells can bind to TNF-α receptor 1 on neurons and induce neuronal apoptosis. Gp120 also can bind to CXCR4 on neurons to induce apoptosis by promotingcalcium influx and glutamate uptake (Hesselgesser et al., 1998). In the intrinsic pathway, gp120 is able to increase the expression and phosphorylation of p53 and subsequently induce the disruption of the mitochondrial membrane by activating BCL-2-associated X protein (Bax; Gougeon, 2003). Interestingly, accumulation ofp53 has been observed in the neurons of AIDS patients (Silva et al., 2003). Gp120 also can activate phospholipase A2 and increase the release of arachidonic acid to disrupt glutamate metabolism in neurons via NMDA-receptor-mediated neurotoxicity, which can lead to cell dysfunction or death (Ushijima et al., 1995).

Mounting evidence indicates that chronic opiate use exacerbates the neuronal damage induced by HIV proteins gp120 and tat through synergy of neuronal apoptosis (Gurwell et al., 2001; Hu et al., 2005) and alteration of dendritic spines and dendrites (Fitting et al., 2010). Ion channels have been implicated in the synergic neurotoxic effects of opioids and gp120. Gp120 can induce K^+^ eﬄux by activating K channels when it binds to CXCR4 (Chen et al., 2011a). Chronic administration of opioids can reduce K^+^ inflow by down-regulating MOR (Christie, 2008). Thus, dysfunction of K^+^ channels may contribute to neurotoxicity and may be a point of convergence for gp120-opioid synergism of chronic pain (Podhaizer et al., 2012). In addition, chemokine receptors can dimerize with MOR, implying a functional interaction between these receptors (Toth et al., 2004). In this context, CXCR4 and CCR5 are particularly interesting because they not only are co-receptors of gp120 but are also co-expressed with MOR on neurons (Sengupta et al., 2009; Heinisch et al., 2011). Besides synergism in neurons, gp120 and opioids may also functionally interact in glia to indirectly facilitate neuronal damage by regulating chemokines (Mahajan et al., 2005) and glutamate uptake (Podhaizer et al., 2012), which is the focus of a later section.

When gp120 and/or chronic opioids cause damage to pain-processing neurons, they may induce neuropathic pain. Gp120 not only directly excites rat DRG neurons and induces allodynia by activating their chemokine receptors (Oh et al., 2001) but also mediates local axonal degeneration of cultured rodent DRG neurons, which is dependent on activation of the caspase pathways (Melli et al., 2006). By a similar mechanism, exposure to gp120 also leads to up-regulation of MCP-1 and CCR2 on DRG neurons and upregulation of their activation, which is expected to contribute to neuropathic pain (Miller et al., 2009). We have generated a gp120 neuropathic pain model that develops similar neuropathologies as human HIV patients, including peripheral neuropathy and synapse degeneration (Yuan et al., 2014).

Accumulating data indicate that glial-neuronal cross-talk plays an important role in opioid-abuse-induced paradoxical pain (Raghavendra et al., 2003; Hutchinson et al., 2008a; Zhao et al., 2012; Sun et al., 2014). The underlying mechanism remains obscure. Application of exogenous CXCL12 (Heinisch et al., 2011) and gp120 (Chen et al., 2011b), both of which are ligands of CXCR4 receptor, significantly attenuate morphine-mediated hyperpolarization in rodent periaqueductal grey (PAG) neurons. Wilson et al. (2011) also suggested that sensory neurons sensitized by the CXCL12-CXCR4 axis (or the gp120-CXCR4 axis) may facilitate the hyperalgesia induced by opioids. Moreover, morphine induces CXCR4-mediated activation of extracellular signal-regulated kinase (ERK) in rat neurons, a crucial regulator of peripheral and central sensitization. This may be one mechanism of gp120/opioid synergism in neuropathic pain (Ji, 2004; Patel et al., 2006; Sengupta et al., 2009).

## Glial Mechanisms

Converging evidence indicates a key role for glia and neuron-glia interactions in the development and maintenance of neuropathic pain (Jin et al., 2003; Hansen and Malcangio, 2013; Ji et al., 2013; Walters, 2014). Glia mediate many of the neurotoxic effects of HIV-1 and co-exposure of glia to opioids and HIV-1 appears to exacerbate proinflammatory and excitotoxic events that lead to neuron dysfunction. Because of these observations, we are investigating the potential that glia-mediated mechanisms may contribute to the pathogenesis of neuropathic pain that is caused by HIV-1 and chronic opioid use.

### Glial Activation and Pain

Glial cells, including astrocytes, microglia, and oligodendrocytes, play important roles in supporting and regulating neuronal functions (Watkins et al., 2005). The involvement of glia in neuropathic pain was first suggested in the mid-1990s (Colburn et al., 1997). Numerous studies have since suggested critical roles of both microglia (Raghavendra et al., 2003; Tsuda et al., 2003; Coull et al., 2005; Ji and Suter, 2007) and astrocytes (Meller et al., 1994; Watkins et al., 1997; Ji et al., 2006; Chiang et al., 2007, 2012; Guo et al., 2007; Okada-Ogawa et al., 2009; Gao and Ji, 2010b; Ren and Dubner, 2010) in the pathogenesis of pathological pain. Activation of astrocytes and microglia can cause neuroanatomical and neurochemical transformations in the CNS that contribute to neuropathic pain (Colburn et al., 1999; Woolf and Mannion, 1999). Malfunctioning astrocytes and microglia may dysregulate synaptic function and neuronal excitability by various mechanisms (Halassa et al., 2007; Pocock and Kettenmann, 2007).

Reactive glia secret proinflammatorycytokines such as tumor necrosis factor-α(TNF-α), IL-1β, and IL-6 that facilitate the expression of central sensitization (Seifert and Maihofner, 2011). Inhibition of the cytokines can effectively reduce neuropathic pain (Moalem and Tracey, 2006). Cytokines are up-regulated in the spinal cord after nerve injury, inflammation, bone cancer, and chronic opioid exposure, and they contribute to the development and maintenance of various types of chronic pain (DeLeo and Yezierski, 2001; Sommer et al., 2001; Watkins et al., 2001; Svensson et al., 2005). For instance, peripheral nerve injury causes the up-regulation of TNF-α and TNFR1 in DRG and the spinal dorsal horn (Schafers et al., 2003; Ohtori et al., 2004; Xu et al., 2006), which facilitates the development of neuropathic pain (Sommer and Kress, 2004; Wieseler-Frank et al., 2005). Inhibition of TNF-α inhibits the pain pathogenesis (George et al., 2000; Ribeiro et al., 2000). IL-1β is induced in the spinal cord in animal models of bone cancer pain, inflammatory pain, and nerve injury pain (Zhang et al., 2005; Guo et al., 2007; Wei et al., 2008; Weyerbacher et al., 2010). Inhibition of spinal IL-1β signaling with IL-1 receptor antagonist (IL-1ra) or neutralizing antibody alleviatespain behaviors (Milligan et al., 2001, 2003; Sweitzer et al., 2001; Guo et al., 2007; Kawasaki et al., 2008b; Wei et al., 2008; Zhang et al., 2008). Conversely, intrathecal injection of IL-1βinduces hyperalgesia (Tadano et al., 1999; Reeve et al., 2000; Ji et al., 2002; Sung et al., 2004; Kawasaki et al., 2008a). Persistent IL-6 increase after spinal cord injury (SCI) appears to correlate with the development of chronic pain both in SCI patients (Davies et al., 2007) and in an animal model of SCI (Detloff et al., 2008). Furthermore, injection of IL-6 in rats causes hypersensitivity to thermal and mechanical stimuli (Oka et al., 1995; Poole et al., 1995; DeLeo et al., 1996; Brenn et al., 2007). Pro-inflammatory cytokines (TNF-α, IL-1β, and IL-6) have been shown to induce the trafficking and surface expression of AMPA receptors in hippocampal neurons (Beattie et al., 2002; Stellwagen et al., 2005), enhance NMDA currents of spinal lamina II neurons (Kawasaki et al., 2008b; Gao et al., 2009), and increase the frequency and amplitude of spontaneous postsynaptic currents (sEPSCs) in dorsal horn neurons (Kawasaki et al., 2008b). These neuronal effects of cytokines may directly contribute to the expression of pathological pain.

Chemokines are a group of cytokines that induce cell migration (Walz et al., 1987; Yoshimura et al., 1987). Recent evidence suggests that chemokine signaling contributes to the pathogenesis of chronic pain by regulating glial activation and neural plasticity (White et al., 2007; Abbadie et al., 2009; Gao and Ji, 2010a; Clark et al., 2011). Among them, CCL2 (MCP-1) is one of the best studied chemokines in pain modulation. It is highly expressed in astrocytes after spinal nerve ligation (Gao et al., 2009) and spinal cord contusion injuries (Knerlich-Lukoschus et al., 2008). Spinal injection of TNF-α-activated astrocytes results in persistent mechanical allodynia by releasing CCL2 (Gao et al., 2010). Chemokines may regulate pain transmission by stimulating specific chemokine receptors such as CCR2, CCR5, CXCR4, and CX3CR1 that are expressed in primary afferent neurons or secondary neurons in the spinal dorsal horn (Abbadie et al., 2003). In spinal cord slices, chemokines were shown to evoke excitatory postsynaptic currents (EPSCs) from lamina II neurons (Yoshimura and Jessell, 1989). Addition of CCL2 to cultured DRG neurons elicits release of calcitonin gene-related peptide (CGRP), a nociceptor neurotransmitter (White et al., 2009).

Glia may also regulate pain pathogenesis by modulating the level of extracellular glutamate, the major excitatory neurotransmitter. Glial glutamate transporter 1 (GLT-1) is abundantly expressed in astrocytes (Beart and O’Shea, 2007). It plays a critical role in clearing extracellular glutamate from synaptic clefts (Huang and Bergles, 2004; Tawfik et al., 2006) and hence modulates glutamatergic transmission and neuronal plasticity (Rothstein et al., 1994, 1996). Inhibition of glutamate transporters results in elevation of spinal extracellular glutamate and spontaneous pain (Liaw et al., 2005; Weng et al., 2006). Spinal nerve injury induces an initial increase (Sung et al., 2003; Wang et al., 2008) followed by a persistent decrease of GLT1 in the spinal cord astrocytes (Tawfik et al., 2008; Xin et al., 2009). GLT-1 gene delivery to the spinal cord attenuates inflammatory and neuropathic pain (Maeda et al., 2008), supporting a critical contribution of glutamate transporter down-regulation to pain pathogenesis (Sung et al., 2003; Weng et al., 2005).

The evidence outlined above illustrates that activated glia may promote pain pathogenesisthrough diverse mechanisms, including releasing pro-inflammatory cytokines and chemokines and down-regulating glutamate transporters. Interestingly, as we will discuss in the next sections, emerging evidence indicates that HIV-1 infection and chronic opioid use also dysregulate these pain pathogenic processes.

### HIV-1 and Opioids in Glial Activation

Opiate drug abuse and HIV-1 are interlinked epidemics (Bell et al., 1998; Anthony et al., 2008), and opioids can exacerbate the neuropathogenesis of HIV-1 (Hauser et al., 2012). In human HIV-1 patients, opiate drug abuse was reported to increases glial reactivity in the CNS with specific alterations in the number and morphology of reactive microglia (Bell et al., 2002). Similarly, morphine rapidly and significantly increases the activation of microglia in the brains of Tat transgenic mice. Additionally, both HIV-1 proteins (e.g., gp120 and Tat) and opioids can activate astrocytes in the SDH (Milligan et al., 2001; Huang et al., 2012).

Emerging evidence supports a role for the interaction of opioids and HIV viral proteins in glial activation, although the underlying mechanisms remain unclear. The interaction depends on mu-opioid receptors (Zou et al., 2011), which are widely expressed in astrocytes (Stiene-Martin et al., 1998, 2001) and microglia (Tomassini et al., 2004). While opioids can directly cause glial activation (El-Hage et al., 2005, 2006, 2008a,b; Bruce-Keller et al., 2008; Turchan-Cholewo et al., 2008, 2009; Gupta et al., 2010), intrastriatal Tat infusion enhanced the activation of glia *in vivo* (El-Hage et al., 2006). Morphine analgesics can activate both classical opioid receptors andthe non-classical receptor Toll-like receptor 4 (TLR4; Hutchinson et al., 2010b), which is expressed in glia that are implicated in various chronic pain syndromes (Hutchinson et al., 2008b, 2010a; Lewis et al., 2010, 2012). Additional evidence indicates that TLR4 activation opposes the analgesic effect of morphine (Watkins et al., 2009; Hutchinson et al., 2010b). The type of opioid receptor that is involved in the opioid/HIV-1 interaction for pain-related synergistic activation of glia is unknown.

Intracellular calcium may be a critical mediator in astrocyte activation that is induced by HIV-1 protein and opioids. Tat or gp120 can evoke an increase in intracellular Ca^2+^ ([Ca^2+^]i) in astroglia (Haughey et al., 1999; Holden et al., 1999). Similar effects are also observed after acute μ-opioid receptor activation (Hauser et al., 1998). Morphine and HIV-1 viral proteins synergistically induce Ca^2+^release from the endoplasmic reticulum (ER) and Ca^2+^ influx from extracellular spaces of astrocytes, which enhance cytokine and chemokine release (El-Hage et al., 2008b). The increased [Ca^2+^]i may contribute to the development of hyperalgesia by regulating synaptic transmission and activity of NMDA and AMPA receptors in the spinal cord (Meller et al., 1996; Guo et al., 2007; Chen et al., 2010b). Furthermore, increased intracellular Ca^2+^ can also activate Ca^2+^-sensitive proteins such as protein kinase C (PKC) and calcium/calmodulin-dependent protein kinase II (CaMK II; Kuhl et al., 2000), both of which play crucial roles in central sensitization during the development of neuropathic and inflammatory pain (Malmberg et al., 1997; Martin et al., 2001; Chen et al., 2010a). CaMKIIα is required for the initiation and maintenance of opioid-induced hyperalgesia (Chen et al., 2010a). Together, these findings indicate that enhanced intracellular Ca^2+^ might be vital for astrocyte activation during pain development. Opioids may synergize with HIV viral proteins in these processes in glial cells. As a result, normally neuroprotective glia (Kaul et al., 2001) and mononuclear phagocytes (Persidsky and Gendelman, 2003) are functionally transformed into deleterious states thatdisrupt CNS homeostasis and create pathophysiological conditions that induce in juries in pain-processing neurons.

### Interactions of Opioids and HIV-1 in Neuropathic Pain

Findings such as those summarized above indicate that the interactions of HIV-1 and opioids have a synergistic effect on glial activation. Since glial activation plays a key role in neuropathic pain development, we reason that HIV-1 infection and opioids interact to promote pain pathogenesis. Several pathogenic pathways can be envisioned to mediate the synergistic effect of opioids and HIV-1 proteins in this scenario.

One of the potential mechanisms is probably mediated by the enhanced pro-inflammatory cytokine release from activated glia. Glial cells are the major source of cytokines (e.g., TNF-α, IL-1β, and IL-6) in the HIV-1-infected CNS (Dong and Benveniste, 2001; Luo et al., 2003). Opioids exacerbate the glial response to HIV-1by accelerating cytokine release (El-Hage et al., 2005). Additionally, HIV replication in microglia can be stimulated by opioids, which leads to the release of toxic viral proteins that then stimulate the release of inflammatory toxins (Glass et al., 1995; Nath et al., 1999; Yadav and Collman, 2009). Opioids may directly activate MOR on microglia (Bruce-Keller et al., 2008; El-Hage et al., 2008a; Turchan-Cholewo et al., 2008, 2009; Gupta et al., 2010) to evoke cytokine and reactive/oxidative responses to insults (Wetzel et al., 2000; Rahim et al., 2003; Qin et al., 2005; Wang et al., 2005). NF-κB is involved in the induction of cytokines in glia (Zhai et al., 2004). HIV-1 Tat activates NF-κB (Conant et al., 1996; El-Hage et al., 2008a) to cause the release of a large amount of cytokines by glia (Conant et al., 1998; El-Hage et al., 2005, 2006, 2008a). Pro-inflammatory cytokines could facilitate the development of hyperalgesia by regulating the activity of synaptic receptors such as NMDARs and AMPARs (Meller et al., 1996; Guo et al., 2007; Chen et al., 2010b). For instance, IL-1β, IL-6, and TNF-α can enhance excitatory synaptic transmission and increased density and conductance of neuronal AMPA (Ogoshi et al., 2005; Stellwagen et al., 2005) and NMDA (Viviani et al., 2003) receptors, and these cytokines can down-regulate neuronal GABA receptors (Stellwagen et al., 2005).

Glia are also a major source of chemokines (e.g., CCL2/MCP-1, CCL5/RANTES, and CCL3/MIP-1a) in the HIV-1-infected CNS (Dong and Benveniste, 2001; Luo et al., 2003). The contribution of chemokines to pain pathogenesis is well established (Liou et al., 2013). Opioids exacerbate the astroglial response to HIV-1and stimulate release of chemokines (El-Hage et al., 2005). NF-κB signaling is also implicated in chemokine induction in astrocytes (Zhai et al., 2004). HIV-1Tat activates NF-κB (Conant et al., 1996; El-Hage et al., 2008a) to elicit release of many chemokines from astroglia (Conant et al., 1998; El-Hage et al., 2005, 2006, 2008a). Previous studies observed synergistic effects of HIV-1 viral proteins and opiate agonists on chemokine release from glia (El-Hage et al., 2006). Similar to the cytokines discussed above, chemokines may also promote the development of hyperalgesia by regulating synaptic transmission and activity of NMDA and AMPA receptors (Meller et al., 1996; Guo et al., 2007; Chen et al., 2010b). Since specific chemokine receptors such as CCR2, CCR5, CXCR4, and CX3CR1 are expressed in primary afferents and/or secondary neurons in the spinal dorsal horn (Oh et al., 2001; Abbadie, 2005), chemokines may modulate pain signal transmission.

Another potential mechanism by which opioids and HIV-1 may cooperate to dysregulate pain-related glial function is to down-regulate their glutamate re-uptake activity. Under normal physiologic conditions, extracellular glutamate is rapidly cleaned by re-uptake processes mediated by glutamate transporters EAAT1 (a.k.a. GLAST) and EAAT2 (a.k.a. GLT-1). EAAT1 and EAAT2 are predominantly expressed by astroglia (but only have low expression in microglia; Gras et al., 2006, 2012). Chronic morphine administration induces down-regulation of spinal glutamate transporters (Ozawa et al., 2001; Mao et al., 2002b). Interestingly, a decrease of EAAT2 expression was also observed in human astrocytes exposed to gp120 or HIV-1 (Wang et al., 2003). Exposure to morphine and HIV-1 Tat boosts extracellular glutamate accumulation at the synapse (Madl and Burgesser, 1993; Phillis et al., 2000), which is expected to cause excitotoxicity (Albrecht et al., 2010).

The above findings indicate that opioids and HIV-1 interact to activate glia, leading to enhanced cytokine and chemokine expression (Mahajan et al., 2005) and down-regulation of glutamate uptake (Zou et al., 2011). These biological effects are detrimental to neurons and may directly contribute to the development of hyperalgesia.

## Summary

Opioid abuse and HIV-1 have been described as interrelated epidemics, and they can exacerbate the neuropathogenesis of neuroAIDS (Hauser et al., 2012). Here, we have mainly considered the potential mechanisms by which the interaction of opioids and HIV-1 facilitates the development of hyperalgesia. Because mounting evidence suggests that they have synergistic effects on neurons and glia, we are working to understand the pathogenic processes from the perspectives of these cell types and their interactions. Based on the previously findings discussed earlier, we suggest that HIV-1 and opioids dysregulate the function of neurons and glia in the pain-processing neural pathway. The ongoing reactive cross-talk between opiate drugs and HIV-1 may not only directly injure pain-transmission neurons but also indirectly contribute to the injuries by activating glia, especially microglia and astrocytes.

## Author Contributions

All authors listed, have made substantial, direct and intellectual contribution to the work, and approved it for publication.

## Conflict of Interest Statement

The authors declare that the research was conducted in the absence of any commercial or financial relationships that could be construed as a potential conflict of interest.

## References

[B1] Abbadie C. (2005). Chemokines, chemokine receptors and pain. *Trends Immunol.* 26 529–534. 10.1016/j.it.2005.08.001 16099720

[B2] Abbadie C. Bhangoo S. De Koninck Y. Malcangio M. Melik-Parsadaniantz S. White F. A. (2009). Chemokines and pain mechanisms. *Brain Res. Rev.* 60 125–134. 10.1016/j.brainresrev.2008.12.002 19146875 PMC2691997

[B3] Abbadie C. Lindia J. A. Cumiskey A. M. Peterson L. B. Mudgett J. S. Bayne E. K. (2003). Impaired neuropathic pain responses in mice lacking the chemokine receptor CCR2. *Proc. Natl. Acad. Sci. U.S.A.* 100 7947–7952. 10.1073/pnas.1331358100 12808141 PMC164693

[B4] Albrecht J. Sidoryk-Wegrzynowicz M. Zielinska M. Aschner M. (2010). Roles of glutamine in neurotransmission. *Neuron Glia Biol.* 6 263–276. 10.1017/S1740925X11000093 22018046

[B5] Anthony I. C. Arango J. C. Stephens B. Simmonds P. Bell J. E. (2008). The effects of illicit drugs on the HIV infected brain. *Front. Biosci.* 13:1294–1307. 10.2741/2762 17981630

[B6] Anthony I. C. Ramage S. N. Carnie F. W. Simmonds P. Bell J. E. (2005). Does drug abuse alter microglial phenotype and cell turnover in the context of advancing HIV infection? *Neuropathol. Appl. Neurobiol*. 31 325–338. 10.1111/j.1365-2990.2005.00648.x 15885069

[B7] Aouizerat B. E. Miaskowski C. A. Gay C. Portillo C. J. Coggins T. Davis H. (2010). Risk factors and symptoms associated with pain in HIV-infected adults. *J. Assoc. Nurses AIDS Care* 21 125–133. 10.1016/j.jana.2009.10.003 20116299 PMC2832225

[B8] Arenas-Pinto A. Bhaskaran K. Dunn D. Weller I. V. (2008). The risk of developing peripheral neuropathy induced by nucleoside reverse transcriptase inhibitors decreases over time: evidence from the Delta trial. *Antivir. Ther.* 13 289–295. 18505180

[B9] Bagetta G. Corasaniti M. T. Berliocchi L. Nistico R. Giammarioli A. M. Malorni W. (1999). Involvement of interleukin-1beta in the mechanism of human immunodeficiency virus type 1 (HIV-1) recombinant protein gp120-induced apoptosis in the neocortex of rat. *Neuroscience* 89 1051–1066. 10.1016/S0306-4522(98)00363-7 10362294

[B10] Baron R. (2000). Peripheral neuropathic pain: from mechanisms to symptoms. *Clin. J. Pain* 16 S12–S20. 10.1097/00002508-200006001-00004 10870735

[B11] Baron R. Levine J. D. Fields H. L. (1999). Causalgia and reflex sympathetic dystrophy: does the sympathetic nervous system contribute to the generation of pain? *Muscle Nerve* 22 678–695. 10.1002/(SICI)1097-4598(199906)22:6<678::AID-MUS4>3.0.CO;2-P 10366221

[B12] Basu S. Bruce R. D. Barry D. T. Altice F. L. (2007). Pharmacological pain control for human immunodeficiency virus-infected adults with a history of drug dependence. *J. Subst. Abuse Treat.* 32 399–409. 10.1016/j.jsat.2006.10.005 17481463 PMC2128698

[B13] Beart P. M. O’Shea R. D. (2007). Transporters for L-glutamate: an update on their molecular pharmacology and pathological involvement. *Br. J. Pharmacol.* 150 5–17. 10.1038/sj.bjp.0706949 17088867 PMC2013845

[B14] Beattie E. C. Stellwagen D. Morishita W. Bresnahan J. C. Ha B. K. Von Zastrow M. (2002). Control of synaptic strength by glial TNFalpha. *Science* 295 2282–2285. 10.1126/science.1067859 11910117

[B15] Bell J. E. Arango J. C. Anthony I. C. (2006). Neurobiology of multiple insults: HIV-1-associated brain disorders in those who use illicit drugs. *J. Neuroimmune Pharmacol.* 1 182–191. 10.1007/s11481-006-9018-2 18040783

[B16] Bell J. E. Arango J. C. Robertson R. Brettle R. P. Leen C. Simmonds P. (2002). HIV and drug misuse in the Edinburgh cohort. *J. Acquir. Immune defic. Syndr.* 31(Suppl. 2) S35–S42. 10.1097/00126334-200210012-00003 12394781

[B17] Bell J. E. Brettle R. P. Chiswick A. Simmonds P. (1998). HIV encephalitis, proviral load and dementia in drug users and homosexuals with AIDS. Effect of neocortical involvement. *Brain* 121(Pt 11) 2043–2052. 10.1093/brain/121.11.2043 9827765

[B18] Bennett B. A. Rusyniak D. E. Hollingsworth C. K. (1995). HIV-1 gp120-induced neurotoxicity to midbrain dopamine cultures. *Brain Res.* 705 168–176. 10.1016/0006-8993(95)01166-8 8821747

[B19] Brenn D. Richter F. Schaible H. G. (2007). Sensitization of unmyelinated sensory fibers of the joint nerve to mechanical stimuli by interleukin-6 in the rat: an inflammatory mechanism of joint pain. *Arthritis Rheum.* 56 351–359. 10.1002/art.22282 17195239

[B20] Bruce-Keller A. J. Turchan-Cholewo J. Smart E. J. Geurin T. Chauhan A. Reid R. (2008). Morphine causes rapid increases in glial activation and neuronal injury in the striatum of inducible HIV-1 Tat transgenic mice. *Glia* 56 1414–1427. 10.1002/glia.20708 18551626 PMC2725184

[B21] Campana W. M. Myers R. R. (2003). Exogenous erythropoietin protects against dorsal root ganglion apoptosis and pain following peripheral nerve injury. *Eur. J. Neurosci.* 18 1497–1506. 10.1046/j.1460-9568.2003.02875.x 14511329

[B22] Chen L. Liu J. Xu C. Keblesh J. Zang W. Xiong H. (2011a). HIV-1gp120 induces neuronal apoptosis through enhancement of 4-aminopyridine-senstive outward K+ currents. *PLoS ONE* 6:e25994 10.1371/journal.pone.0025994 PMC3189248 22016798

[B23] Chen X. Kirby L. G. Palma J. Benamar K. Geller E. B. Eisenstein T. K. (2011b). The effect of gp120 on morphine’s antinociceptive and neurophysiological actions. *Brain Behav. Immun.* 25 1434–1443. 10.1016/j.bbi.2011.04.014 21569838 PMC3998826

[B24] Chen W. Tang Z. Fortina P. Patel P. Addya S. Surrey S. (2005). Ethanol potentiates HIV-1 gp120-induced apoptosis in human neurons via both the death receptor and NMDA receptor pathways. *Virology* 334 59–73. 10.1016/j.virol.2005.01.014 15749123

[B25] Chen Y. Yang C. Wang Z. J. (2010a). Ca2+/calmodulin-dependent protein kinase II alpha is required for the initiation and maintenance of opioid-induced hyperalgesia. *J. Neurosci.* 30 38–46. 10.1523/JNEUROSCI.4346-09.2010 20053885 PMC2821163

[B26] Chen Z. Muscoli C. Doyle T. Bryant L. Cuzzocrea S. Mollace V. (2010b). NMDA-receptor activation and nitroxidative regulation of the glutamatergic pathway during nociceptive processing. *Pain* 149 100–106. 10.1016/j.pain.2010.01.015 20167432 PMC2837917

[B27] Chiang C. Y. Sessle B. J. Dostrovsky J. O. (2012). Role of astrocytes in pain. *Neurochem. Res.* 37 2419–2431. 10.1007/s11064-012-0801-6 22638776

[B28] Chiang C. Y. Wang J. Xie Y. F. Zhang S. Hu J. W. Dostrovsky J. O. (2007). Astroglial glutamate-glutamine shuttle is involved in central sensitization of nociceptive neurons in rat medullary dorsal horn. *J. Neurosci.* 27 9068–9076. 10.1523/JNEUROSCI.2260-07.2007 17715343 PMC6672204

[B29] Christie M. J. (2008). Cellular neuroadaptations to chronic opioids: tolerance, withdrawal and addiction. *Br. J. Pharmacol.* 154 384–396. 10.1038/bjp.2008.100 18414400 PMC2442443

[B30] Clark A. K. Staniland A. A. Malcangio M. (2011). Fractalkine/CX3CR1 signalling in chronic pain and inflammation. *Curr. Pharm. Biotechnol.* 12 1707–1714. 10.2174/138920111798357465 21466443

[B31] Colburn R. W. DeLeo J. A. Rickman A. J. Yeager M. P. Kwon P. Hickey W. F. (1997). Dissociation of microglial activation and neuropathic pain behaviors following peripheral nerve injury in the rat. *J. Neuroimmunol.* 79 163–175. 10.1016/S0165-5728(97)00119-7 9394789

[B32] Colburn R. W. Rickman A. J. DeLeo J. A. (1999). The effect of site and type of nerve injury on spinal glial activation and neuropathic pain behavior. *Exp. Neurol.* 157 289–304. 10.1006/exnr.1999.7065 10364441

[B33] Conant K. Garzino-Demo A. Nath A. McArthur J. C. Halliday W. Power C. (1998). Induction of monocyte chemoattractant protein-1 in HIV-1 Tat-stimulated astrocytes and elevation in AIDS dementia. *Proc. Natl. Acad. Sci. U.S.A.* 95 3117–3121. 10.1073/pnas.95.6.3117 9501225 PMC19704

[B34] Conant K. Ma M. Nath A. Major E. O. (1996). Extracellular human immunodeficiency virus type 1 Tat protein is associated with an increase in both NF-kappa B binding and protein kinase C activity in primary human astrocytes. *J. Virol.* 70 1384–1389. 8627654 10.1128/jvi.70.3.1384-1389.1996 PMC189957

[B35] Corasaniti M. T. Bilotta A. Strongoli M. C. Navarra M. Bagetta G. Di Renzo G. (2001a). HIV-1 coat protein gp120 stimulates interleukin-1beta secretion from human neuroblastoma cells: evidence for a role in the mechanism of cell death. *Br. J. Pharmacol.* 134 1344–1350. 10.1038/sj.bjp.0704382 11704656 PMC1573068

[B36] Corasaniti M. T. Piccirilli S. Paoletti A. Nistico R. Stringaro A. Malorni W. (2001b). Evidence that the HIV-1 coat protein gp120 causes neuronal apoptosis in the neocortex of rat via a mechanism involving CXCR4 chemokine receptor. *Neurosci. Lett.* 312 67–70. 10.1016/S0304-3940(01)02191-7 11595336

[B37] Cornblath D. R. McArthur J. C. (1988). Predominantly sensory neuropathy in patients with AIDS and AIDS-related complex. *Neurology* 38 794–796. 10.1212/WNL.38.5.794 2834669

[B38] Coull J. A. Beggs S. Boudreau D. Boivin D. Tsuda M. Inoue K. (2005). BDNF from microglia causes the shift in neuronal anion gradient underlying neuropathic pain. *Nature* 438 1017–1021. 10.1038/nature04223 16355225

[B39] Coull J. A. Boudreau D. Bachand K. Prescott S. A. Nault F. Sik A. (2003). Trans-synaptic shift in anion gradient in spinal lamina I neurons as a mechanism of neuropathic pain. *Nature* 424 938–942. 10.1038/nature01868 12931188

[B40] Davies A. L. Hayes K. C. Dekaban G. A. (2007). Clinical correlates of elevated serum concentrations of cytokines and autoantibodies in patients with spinal cord injury. *Arch. Phys. Med. Rehabil.* 88 1384–1393. 10.1016/j.apmr.2007.08.004 17964877

[B41] DeLeo J. A. Colburn R. W. Nichols M. Malhotra A. (1996). Interleukin-6-mediated hyperalgesia/allodynia and increased spinal IL-6 expression in a rat mononeuropathy model. *J. Interferon Cytokine Res.* 16 695–700. 10.1089/jir.1996.16.695 8887053

[B42] DeLeo J. A. Yezierski R. P. (2001). The role of neuroinflammation and neuroimmune activation in persistent pain. *Pain* 90 1–6. 10.1016/S0304-3959(00)00490-5 11166964

[B43] Detloff M. R. Fisher L. C. McGaughy V. Longbrake E. E. Popovich P. G. Basso D. M. (2008). Remote activation of microglia and pro-inflammatory cytokines predict the onset and severity of below-level neuropathic pain after spinal cord injury in rats. *Exp. Neurol.* 212 337–347. 10.1016/j.expneurol.2008.04.009 18511041 PMC2600773

[B44] Dong Y. Benveniste E. N. (2001). Immune function of astrocytes. *Glia* 36 180–190. 10.1002/glia.1107 11596126

[B45] El-Hage N. Bruce-Keller A. J. Knapp P. E. Hauser K. F. (2008a). CCL5/RANTES gene deletion attenuates opioid-induced increases in glial CCL2/MCP-1 immunoreactivity and activation in HIV-1 Tat-exposed mice. *J. Neuroimmune Pharmacol.* 3 275–285. 10.1007/s11481-008-9127-1 18815890 PMC2722754

[B46] El-Hage N. Bruce-Keller A. J. Yakovleva T. Bazov I. Bakalkin G. Knapp P. E. (2008b). Morphine exacerbates HIV-1 Tat-induced cytokine production in astrocytes through convergent effects on [Ca(2+)](i), NF-kappaB trafficking and transcription. *PLoS ONE* 3:e4093 10.1371/journal.pone.0004093 PMC2605563 19116667

[B47] El-Hage N. Gurwell J. A. Singh I. N. Knapp P. E. Nath A. Hauser K. F. (2005). Synergistic increases in intracellular Ca2(+, and the release of MCP-1, RANTES, and IL-6 by astrocytes treated with opiates and HIV-1 Tat. *Glia* 50 91–106. 10.1002/glia.20148 15630704 PMC4301446

[B48] El-Hage N. Wu G. Wang J. Ambati J. Knapp P. E. Reed J. L. (2006). HIV-1 Tat and opiate-induced changes in astrocytes promote chemotaxis of microglia through the expression of MCP-1 and alternative chemokines. *Glia* 53 132–146. 10.1002/glia.20262 16206161 PMC3077280

[B49] Elmore S. (2007). Apoptosis: a review of programmed cell death. *Toxicol. Pathol.* 35 495–516. 10.1080/01926230701320337 17562483 PMC2117903

[B50] Evers S. Wibbeke B. Reichelt D. Suhr B. Brilla R. Husstedt I. (2000). The impact of HIV infection on primary headache. Unexpected findings from retrospective, cross-sectional, and prospective analyses. *Pain* 85 191–200. 10.1016/S0304-3959(99)00266-3 10692618

[B51] Fitting S. Xu R. Bull C. Buch S. K. El-Hage N. Nath A. (2010). Interactive comorbidity between opioid drug abuse and HIV-1 Tat: chronic exposure augments spine loss and sublethal dendritic pathology in striatal neurons. *Am. J. Pathol.* 177 1397–1410. 10.2353/ajpath.2010.090945 20651230 PMC2928972

[B52] Gao Y. J. Ji R. R. (2010a). Chemokines, neuronal-glial interactions, and central processing of neuropathic pain. *Pharmacol. Ther.* 126 56–68. 10.1016/j.pharmthera.2010.01.002 20117131 PMC2839017

[B53] Gao Y. J. Ji R. R. (2010b). Targeting astrocyte signaling for chronic pain. *Neurotherapeutics* 7 482–493. 10.1016/j.nurt.2010.05.016 20880510 PMC2950097

[B54] Gao Y. J. Zhang L. Ji R. R. (2010). Spinal injection of TNF-alpha-activated astrocytes produces persistent pain symptom mechanical allodynia by releasing monocyte chemoattractant protein-1. *Glia* 58 1871–1880. 10.1002/glia.21056 20737477 PMC2951489

[B55] Gao Y. J. Zhang L. Samad O. A. Suter M. R. Yasuhiko K. Xu Z. Z. (2009). JNK-induced MCP-1 production in spinal cord astrocytes contributes to central sensitization and neuropathic pain. *J. Neurosci.* 29 4096–4108. 10.1523/JNEUROSCI.3623-08.2009 19339605 PMC2682921

[B56] George A. Marziniak M. Schafers M. Toyka K. V. Sommer C. (2000). Thalidomide treatment in chronic constrictive neuropathy decreases endoneurial tumor necrosis factor-alpha, increases interleukin-10 and has long-term effects on spinal cord dorsal horn met-enkephalin. *Pain* 88 267–275. 10.1016/S0304-3959(00)00333-X 11068114

[B57] Glass J. D. Fedor H. Wesselingh S. L. McArthur J. C. (1995). Immunocytochemical quantitation of human immunodeficiency virus in the brain: correlations with dementia. *Ann. Neurol.* 38 755–762. 10.1002/ana.410380510 7486867

[B58] Gougeon M. L. (2003). Apoptosis as an HIV strategy to escape immune attack. *Nat. Rev. Immunol.* 3 392–404. 10.1038/nri1087 12766761

[B59] Gras G. Porcheray F. Samah B. Leone C. (2006). The glutamate-glutamine cycle as an inducible, protective face of macrophage activation. *J. Leukoc. Biol.* 80 1067–1075. 10.1189/jlb.0306153 16912070

[B60] Gras G. Samah B. Hubert A. Leone C. Porcheray F. Rimaniol A. C. (2012). EAAT expression by macrophages and microglia: still more questions than answers. *Amino Acids* 42 221–229. 10.1007/s00726-011-0866-6 21373769

[B61] Guo W. Wang H. Watanabe M. Shimizu K. Zou S. LaGraize S. C. (2007). Glial-cytokine-neuronal interactions underlying the mechanisms of persistent pain. *J. Neurosci.* 27 6006–6018. 10.1523/JNEUROSCI.0176-07.2007 17537972 PMC2676443

[B62] Gupta S. Knight A. G. Gupta S. Knapp P. E. Hauser K. F. Keller J. N. (2010). HIV-Tat elicits microglial glutamate release: role of NAPDH oxidase and the cystine-glutamate antiporter. *Neurosci. Lett.* 485 233–236. 10.1016/j.neulet.2010.09.019 20849923 PMC2956797

[B63] Gurwell J. A. Nath A. Sun Q. Zhang J. Martin K. M. Chen Y. (2001). Synergistic neurotoxicity of opioids and human immunodeficiency virus-1 Tat protein in striatal neurons in vitro. *Neuroscience* 102 555–563. 10.1016/S0306-4522(00)00461-9 11226693 PMC4300203

[B64] Hains B. C. Saab C. Y. Klein J. P. Craner M. J. Waxman S. G. (2004). Altered sodium channel expression in second-order spinal sensory neurons contributes to pain after peripheral nerve injury. *J. Neurosci.* 24 4832–4839. 10.1523/JNEUROSCI.0300-04.2004 15152043 PMC6729453

[B65] Halassa M. M. Fellin T. Haydon P. G. (2007). The tripartite synapse: roles for gliotransmission in health and disease. *Trends Mol. Med.* 13 54–63. 10.1016/j.molmed.2006.12.005 17207662

[B66] Hansen R. R. Malcangio M. (2013). Astrocytes–multitaskers in chronic pain. *Eur. J. Pharmacol.* 716 120–128. 10.1016/j.ejphar.2013.03.023 23528354

[B67] Hao S. (2013). The molecular and pharmacological mechanisms of HIV-related neuropathic pain. *Curr. Neuropharmacol.* 11 499–512. 10.2174/1570159X11311050005 24403874 PMC3763758

[B68] Haughey N. J. Holden C. P. Nath A. Geiger J. D. (1999). Involvement of inositol 1,4,5-trisphosphate-regulated stores of intracellular calcium in calcium dysregulation and neuron cell death caused by HIV-1 protein tat. *J. Neurochem.* 73 1363–1374. 10.1046/j.1471-4159.1999.0731363.x 10501179

[B69] Hauser K. F. Fitting S. Dever S. M. Podhaizer E. M. Knapp P. E. (2012). Opiate drug use and the pathophysiology of neuroAIDS. *Curr. HIV Res.* 10 435–452. 10.2174/157016212802138779 22591368 PMC3431547

[B70] Hauser K. F. Harris-White M. E. Jackson J. A. Opanashuk L. A. Carney J. M. (1998). Opioids disrupt Ca2+ homeostasis and induce carbonyl oxyradical production in mouse astrocytes in vitro: transient increases and adaptation to sustained exposure. *Exp. Neurol.* 151 70–76. 10.1006/exnr.1998.6788 9582255

[B71] Heinisch S. Palma J. Kirby L. G. (2011). Interactions between chemokine and mu-opioid receptors: anatomical findings and electrophysiological studies in the rat periaqueductal grey. *Brain Behav. Immun.* 25 360–372. 10.1016/j.bbi.2010.10.020 20974247 PMC3025063

[B72] Herzberg U. Sagen J. (2001). Peripheral nerve exposure to HIV viral envelope protein gp120 induces neuropathic pain and spinal gliosis. *J. Neuroimmunol.* 116 29–39. 10.1016/S0165-5728(01)00288-0 11311327

[B73] Hesselgesser J. HalksMiller M. DelVecchio V. Peiper S. C. Hoxie J. Kolson D. L. (1997). CD4-independent association between HIV-1 gp120 and CXCR4: functional chemokine receptors are expressed in human neurons. *Curr. Biol.* 7 112–121. 10.1016/S0960-9822(06)00055-8 9024623

[B74] Hesselgesser J. Taub D. Baskar P. Greenberg M. Hoxie J. Kolson D. L. (1998). Neuronal apoptosis induced by HIV-1 gp120 and the chemokine SDF-1 alpha is mediated by the chemokine receptor CXCR4. *Curr. Biol.* 8 595–598. 10.1016/S0960-9822(98)70230-1 9601645

[B75] Hewitt D. J. McDonald M. Portenoy R. K. Rosenfeld B. Passik S. Breitbart W. (1997). Pain syndromes and etiologies in ambulatory AIDS patients. *Pain* 70 117–123. 10.1016/S0304-3959(96)03281-2 9150284

[B76] Hoke A. Morris M. Haughey N. J. (2009). GPI-1046 protects dorsal root ganglia from gp120-induced axonal injury by modulating store-operated calcium entry. *J. Peripher. Nerv. Syst.* 14 27–35. 10.1111/j.1529-8027.2009.00203.x 19335537 PMC2728770

[B77] Holden C. P. Haughey N. J. Nath A. Geiger J. D. (1999). Role of Na+/H+ exchangers, excitatory amino acid receptors and voltage-operated Ca2+ channels in human immunodeficiency virus type 1 gp120-mediated increases in intracellular Ca2+ in human neurons and astrocytes. *Neuroscience* 91 1369–1378. 10.1016/S0306-4522(98)00714-3 10391443

[B78] Hong S. Wiley J. W. (2005). Early painful diabetic neuropathy is associated with differential changes in the expression and function of vanilloid receptor 1. *J. Biol. Chem.* 280 618–627. 10.1074/jbc.M408500200 15513920

[B79] Horvath R. J. DeLeo J. A. (2009). Morphine enhances microglial migration through modulation of P2X4 receptor signaling. *J. Neurosci.* 29 998–1005. 10.1523/JNEUROSCI.4595-08.2009 19176808 PMC2727471

[B80] Hou Q. Barr T. Gee L. Vickers J. Wymer J. Borsani E. (2011). Keratinocyte expression of calcitonin gene-related peptide beta: implications for neuropathic and inflammatory pain mechanisms. *Pain* 152 2036–2051. 10.1016/j.pain.2011.04.033 21641113 PMC3157543

[B81] Hu S. Sheng W. S. Lokensgard J. R. Peterson P. K. (2005). Morphine potentiates HIV-1 gp120-induced neuronal apoptosis. *J. Infect. Dis.* 191 886–889. 10.1086/427830 15717263

[B82] Huang Y. H. Bergles D. E. (2004). Glutamate transporters bring competition to the synapse. *Curr. Opin. Neurobiol.* 14 346–352. 10.1016/j.conb.2004.05.007 15194115

[B83] Huang Y. N. Tsai R. Y. Lin S. L. Chien C. C. Cherng C. H. Wu C. T. (2012). Amitriptyline attenuates astrocyte activation and morphine tolerance in rats: role of the PSD-95/NR1/nNOS/PKCgamma signaling pathway. *Behav. Brain Res.* 229 401–411. 10.1016/j.bbr.2012.01.044 22309983

[B84] Hudson L. J. Bevan S. Wotherspoon G. Gentry C. Fox A. Winter J. (2001). VR1 protein expression increases in undamaged DRG neurons after partial nerve injury. *Eur. J. Neurosci.* 13 2105–2114. 10.1046/j.0953-816x.2001.01591.x 11422451

[B85] Hutchinson M. R. Lewis S. S. Coats B. D. Rezvani N. Zhang Y. Wieseler J. L. (2010a). Possible involvement of toll-like receptor 4/myeloid differentiation factor-2 activity of opioid inactive isomers causes spinal proinflammation and related behavioral consequences. *Neuroscience* 167 880–893. 10.1016/j.neuroscience.2010.02.011 20178837 PMC2854318

[B86] Hutchinson M. R. Zhang Y. Shridhar M. Evans J. H. Buchanan M. M. Zhao T. X. (2010b). Evidence that opioids may have toll-like receptor 4 and MD-2 effects. *Brain Behav. Immun.* 24 83–95. 10.1016/j.bbi.2009.08.004 19679181 PMC2788078

[B87] Hutchinson M. R. Northcutt A. L. Chao L. W. Kearney J. J. Zhang Y. Berkelhammer D. L. (2008a). Minocycline suppresses morphine-induced respiratory depression, suppresses morphine-induced reward, and enhances systemic morphine-induced analgesia. *Brain Behav. Immun.* 22 1248–1256. 10.1016/j.bbi.2008.07.008 18706994 PMC2783326

[B88] Hutchinson M. R. Zhang Y. Brown K. Coats B. D. Shridhar M. Sholar P. W. (2008b). Non-stereoselective reversal of neuropathic pain by naloxone and naltrexone: involvement of toll-like receptor 4 (TLR4). *Eur. J. Neurosci.* 28 20–29. 10.1111/j.1460-9568.2008.06321.x 18662331 PMC2588470

[B89] Ji G. C. Zhang Y. Q. Ma F. Wu G. C. (2002). Increase of nociceptive threshold induced by intrathecal injection of interleukin-1beta in normal and carrageenan inflammatory rat. *Cytokine* 19 31–36. 10.1006/cyto.2002.1949 12200111

[B90] Ji R. R. (2004). Peripheral and central mechanisms of inflammatory pain, with emphasis on MAP kinases. *Curr. Drug Targets Inflamm. Allergy* 3 299–303. 10.2174/1568010043343804 15379598

[B91] Ji R. R. Berta T. Nedergaard M. (2013). Glia and pain: is chronic pain a gliopathy? *Pain* 154(Suppl. 1) S10–S28. 10.1016/j.pain.2013.06.022 23792284 PMC3858488

[B92] Ji R. R. Kawasaki Y. Zhuang Z. Y. Wen Y. R. Decosterd I. (2006). Possible role of spinal astrocytes in maintaining chronic pain sensitization: review of current evidence with focus on bFGF/JNK pathway. *Neuron Glia Biol.* 2 259–269. 10.1017/S1740925X07000403 17710215 PMC1949390

[B93] Ji R. R. Suter M. R. (2007). p38 MAPK, microglial signaling, and neuropathic pain. *Mol. Pain* 3 33 10.1186/1744-8069-3-33 PMC2186318 17974036

[B94] Ji R. R. Woolf C. J. (2001). Neuronal plasticity and signal transduction in nociceptive neurons: implications for the initiation and maintenance of pathological pain. *Neurobiol. Dis.* 8 1–10. 10.1006/nbdi.2000.0360 11162235

[B95] Jin S. X. Zhuang Z. Y. Woolf C. J. Ji R. R. (2003). p38 mitogen-activated protein kinase is activated after a spinal nerve ligation in spinal cord microglia and dorsal root ganglion neurons and contributes to the generation of neuropathic pain. *J. Neurosci.* 23 4017–4022. 12764087 10.1523/JNEUROSCI.23-10-04017.2003 PMC6741086

[B96] Joseph E. K. Levine J. D. (2004). Caspase signalling in neuropathic and inflammatory pain in the rat. *Eur. J. Neurosci.* 20 2896–2902. 10.1111/j.1460-9568.2004.03750.x 15579143

[B97] Jung H. Miller R. J. (2008). Activation of the nuclear factor of activated T-cells (NFAT) mediates upregulation of CCR2 chemokine receptors in dorsal root ganglion (DRG) neurons: a possible mechanism for activity-dependent transcription in DRG neurons in association with neuropathic pain. *Mol. Cell. Neurosci.* 37 170–177. 10.1016/j.mcn.2007.09.004 17949992 PMC2376051

[B98] Kaul M. Garden G. A. Lipton S. A. (2001). Pathways to neuronal injury and apoptosis in HIV-associated dementia. *Nature* 410 988–994. 10.1038/35073667 11309629

[B99] Kawasaki Y. Xu Z. Z. Wang X. Park J. Y. Zhuang Z. Y. Tan P. H. (2008a). Distinct roles of matrix metalloproteases in the early– and late-phase development of neuropathic pain. *Nat. Med.* 14 331–336. 10.1038/nm1723 18264108 PMC2279180

[B100] Kawasaki Y. Zhang L. Cheng J. K. Ji R. R. (2008b). Cytokine mechanisms of central sensitization: distinct and overlapping role of interleukin-1beta, interleukin-6, and tumor necrosis factor-alpha in regulating synaptic and neuronal activity in the superficial spinal cord. *J. Neurosci.* 28 5189–5194. 10.1523/JNEUROSCI.3338-07.2008 18480275 PMC2408767

[B101] Keswani S. C. Jack C. Zhou C. Hoke A. (2006). Establishment of a rodent model of HIV-associated sensory neuropathy. *J. Neurosci.* 26 10299–10304. 10.1523/JNEUROSCI.3135-06.2006 17021185 PMC6674617

[B102] Knerlich-Lukoschus F. Juraschek M. Blomer U. Lucius R. Mehdorn H. M. Held-Feindt J. (2008). Force-dependent development of neuropathic central pain and time-related CCL2/CCR2 expression after graded spinal cord contusion injuries of the rat. *J. Neurotrauma* 25 427–448. 10.1089/neu.2007.0431 18338959

[B103] Kuhl M. Sheldahl L. C. Park M. Miller J. R. Moon R. T. (2000). The Wnt/Ca2+ pathway: a new vertebrate Wnt signaling pathway takes shape. *Trends Genet.* 16 279–283. 10.1016/s0168-9525(00)02028-x 10858654

[B104] Kumar R. Orsoni S. Norman L. Verma A. S. Tirado G. Giavedoni L. D. (2006). Chronic morphine exposure causes pronounced virus replication in cerebral compartment and accelerated onset of AIDS in SIV/SHIV-infected Indian rhesus macaques. *Virology* 354 192–206. 10.1016/j.virol.2006.06.020 16876224

[B105] Kumar R. Torres C. Yamamura Y. Rodriguez I. Martinez M. Staprans S. (2004). Modulation by morphine of viral set point in rhesus macaques infected with simian immunodeficiency virus and simian-human immunodeficiency virus. *J. Virol.* 78 11425–11428. 10.1128/Jvi.78.20.11425-11428.2004 15452267 PMC521826

[B106] Lai J. Hunter J. C. Porreca F. (2003). The role of voltage-gated sodium channels in neuropathic pain. *Curr. Opin. Neurobiol.* 13 291–297. 10.1016/S0959-4388(03)00074-6 12850213

[B107] Lannuzel A. Lledo P. M. Lamghitnia H. O. Vincent J. D. Tardieu M. (1995). HIV-1 envelope proteins gp120 and gp160 potentiate NMDA-induced [Ca2+]i increase, alter [Ca2+]i homeostasis and induce neurotoxicity in human embryonic neurons. *Eur. J. Neurosci.* 7 2285–2293. 10.1111/j.1460-9568.1995.tb00649.x 8563977

[B108] Lee M. Silverman S. M. Hansen H. Patel V. B. Manchikanti L. (2011). A comprehensive review of opioid-induced hyperalgesia. *Pain Physician* 14 145–161. 21412369

[B109] Lewis S. S. Hutchinson M. R. Rezvani N. Loram L. C. Zhang Y. Maier S. F. (2010). Evidence that intrathecal morphine-3-glucuronide may cause pain enhancement via toll-like receptor 4/MD-2 and interleukin-1beta. *Neuroscience* 165 569–583. 10.1016/j.neuroscience.2009.10.011 19833175 PMC2795035

[B110] Lewis S. S. Loram L. C. Hutchinson M. R. Li C. M. Zhang Y. Maier S. F. (2012). (+)-naloxone, an opioid-inactive toll-like receptor 4 signaling inhibitor, reverses multiple models of chronic neuropathic pain in rats. *J. Pain* 13 498–506. 10.1016/j.jpain.2012.02.005 22520687 PMC3348259

[B111] Liaw W. J. Stephens R. L. Jr. Binns B. C. Chu Y. Sepkuty J. P. Johns R. A. (2005). Spinal glutamate uptake is critical for maintaining normal sensory transmission in rat spinal cord. *Pain* 115 60–70. 10.1016/j.pain.2005.02.006 15836970

[B112] Liou J. T. Lee C. M. Day Y. J. (2013). The immune aspect in neuropathic pain: role of chemokines. *Acta Anaesthesiol. Taiwan.* 51 127–132. 10.1016/j.aat.2013.08.006 24148742

[B113] Lipton S. A. (1992). Memantine prevents HIV coat protein-induced neuronal injury in vitro. *Neurology* 42 1403–1405. 10.1212/WNL.42.7.1403 1620355

[B114] Lipton S. A. (1998). Neuronal injury associated with HIV-1: approaches to treatment. *Annu. Rev. Pharmacol. Toxicol.* 38 159–177. 10.1146/annurev.pharmtox.38.1.159 9597152

[B115] Lipton S. A. Sucher N. J. Kaiser P. K. Dreyer E. B. (1991). Synergistic effects of HIV coat protein and NMDA receptor-mediated neurotoxicity. *Neuron* 7 111–118. 10.1016/0896-6273(91)90079-F 1676893

[B116] Lopez O. L. Becker J. T. Dew M. A. Caldararo R. (2004). Risk modifiers for peripheral sensory neuropathy in HIV infection/AIDS. *Eur. J. Neurol.* 11 97–102. 10.1046/j.1351-5101.2003.00713.x 14748769

[B117] Luciano C. A. Pardo C. A. McArthur J. C. (2003). Recent developments in the HIV neuropathies. *Curr. Opin. Neurol.* 16 403–409. 10.1097/01.wco.0000073943.19076.98 12858079

[B118] Luo Y. Berman M. A. Abromson-Leeman S. R. Dorf M. E. (2003). Tumor necrosis factor is required for RANTES-induced astrocyte monocyte chemoattractant protein-1 production. *Glia* 43 119–127. 10.1002/glia.10231 12838504

[B119] Luo Z. D. Chaplan S. R. Higuera E. S. Sorkin L. S. Stauderman K. A. Williams M. E. (2001). Upregulation of dorsal root ganglion (alpha)2(delta) calcium channel subunit and its correlation with allodynia in spinal nerve-injured rats. *J. Neurosci.* 21 1868–1875. 11245671 10.1523/JNEUROSCI.21-06-01868.2001 PMC6762626

[B120] Madl J. E. Burgesser K. (1993). Adenosine triphosphate depletion reverses sodium-dependent, neuronal uptake of glutamate in rat hippocampal slices. *J. Neurosci.* 13 4429–4444. 8105040 10.1523/JNEUROSCI.13-10-04429.1993 PMC6576387

[B121] Maeda S. Kawamoto A. Yatani Y. Shirakawa H. Nakagawa T. Kaneko S. (2008). Gene transfer of GLT-1, a glial glutamate transporter, into the spinal cord by recombinant adenovirus attenuates inflammatory and neuropathic pain in rats. *Mol. Pain* 4 65 10.1186/1744-8069-4-65 PMC2628654 19108711

[B122] Mahajan S. D. Aalinkeel R. Reynolds J. L. Nair B. B. Fernandez S. F. Schwartz S. A. (2005). Morphine exacerbates HIV-1 viral protein gp120 induced modulation of chemokine gene expression in U373 astrocytoma cells. *Curr. HIV Res.* 3 277–288. 10.2174/1570162054368048 16022659

[B123] Malmberg A. B. Chen C. Tonegawa S. Basbaum A. I. (1997). Preserved acute pain and reduced neuropathic pain in mice lacking PKCgamma. *Science* 278 279–283. 10.1126/science.278.5336.279 9323205

[B124] Mao J. Sung B. Ji R.-R. Lim G. (2002a). Neuronal apoptosis associated with morphine tolerance: evidence for an opioid-induced neurotoxic mechanism. *J. Neurosci.* 22 7650–7661. 12196588 10.1523/JNEUROSCI.22-17-07650.2002 PMC6757968

[B125] Mao J. Sung B. Ji R. R. Lim G. (2002b). Chronic morphine induces downregulation of spinal glutamate transporters: implications in morphine tolerance and abnormal pain sensitivity. *J. Neurosci.* 22 8312–8323. 12223586 10.1523/JNEUROSCI.22-18-08312.2002 PMC6758088

[B126] Marchand F. Perretti M. McMahon S. B. (2005). Role of the immune system in chronic pain. *Nat. Rev. Neurosci.* 6 521–532. 10.1038/nrn1700 15995723

[B127] Martin C. Solders G. Sonnerborg A. Hansson P. (2003). Painful and non-painful neuropathy in HIV-infected patients: an analysis of somatosensory nerve function. *Eur. J. Pain* 7 23–31. 10.1016/S1090-3801(02)00053-8 12527314

[B128] Martin W. J. Malmberg A. B. Basbaum A. I. (2001). PKCgamma contributes to a subset of the NMDA-dependent spinal circuits that underlie injury-induced persistent pain. *J. Neurosci.* 21 5321–5327. 11438608 10.1523/JNEUROSCI.21-14-05321.2001 PMC6762854

[B129] Mattson M. P. Haughey N. J. Nath A. (2005). Cell death in HIV dementia. *Cell Death Differ.* 12(Suppl. 1) 893–904. 10.1038/sj.cdd.4401577 15761472

[B130] Meller S. T. Dykstra C. Gebhart G. F. (1996). Acute mechanical hyperalgesia in the rat can be produced by coactivation of spinal ionotropic AMPA and metabotropic glutamate receptors, activation of phospholipase A2 and generation of cyclooxygenase products. *Prog. Brain Res.* 110 177–192. 10.1016/S0079-6123(08)62574-1 9000725

[B131] Meller S. T. Dykstra C. Grzybycki D. Murphy S. Gebhart G. F. (1994). The possible role of glia in nociceptive processing and hyperalgesia in the spinal cord of the rat. *Neuropharmacology* 33 1471–1478. 10.1016/0028-3908(94)90051-5 7532831

[B132] Melli G. Keswani S. C. Fischer A. Chen W. Hoke A. (2006). Spatially distinct and functionally independent mechanisms of axonal degeneration in a model of HIV-associated sensory neuropathy. *Brain* 129 1330–1338. 10.1093/brain/awl058 16537566

[B133] Meucci O. Miller R. J. (1996). gp120-induced neurotoxicity in hippocampal pyramidal neuron cultures: protective action of TGF-beta1. *J. Neurosci.* 16 4080–4088. 8753870 10.1523/JNEUROSCI.16-13-04080.1996 PMC2689548

[B134] Michaels J. Sharer L. R. Epstein L. G. (1988). Human immunodeficiency virus type 1 (HIV-1) infection of the nervous system: a review. *Immunodefic. Rev.* 1 71–104. 3078711

[B135] Mika J. (2008). Modulation of microglia can attenuate neuropathic pain symptoms and enhance morphine effectiveness. *Pharmacol. Rep.* 60 297–307. 18622054

[B136] Mika J. Wawrzczak-Bargiela A. Osikowicz M. Makuch W. Przewlocka B. (2009). Attenuation of morphine tolerance by minocycline and pentoxifylline in naive and neuropathic mice. *Brain Behav. Immun.* 23 75–84. 10.1016/j.bbi.2008.07.005 18684397

[B137] Miller K. E. Hoffman E. M. Sutharshan M. Schechter R. (2011). Glutamate pharmacology and metabolism in peripheral primary afferents: physiological and pathophysiological mechanisms. *Pharmacol. Ther.* 130 283–309. 10.1016/j.pharmthera.2011.01.005 21276816 PMC5937940

[B138] Miller R. J. Jung H. Bhangoo S. K. White F. A. (2009). Cytokine and chemokine regulation of sensory neuron function. *Handb. Exp. Pharmacol.* 194 417–449. 10.1007/978-3-540-79090-7_12 19655114 PMC2746245

[B139] Milligan E. D. O’Connor K. A. Nguyen K. T. Armstrong C. B. Twining C. Gaykema R. P. (2001). Intrathecal HIV-1 envelope glycoprotein gp120 induces enhanced pain states mediated by spinal cord proinflammatory cytokines. *J. Neurosci.* 21 2808–2819. 11306633 10.1523/JNEUROSCI.21-08-02808.2001 PMC6762530

[B140] Milligan E. D. Twining C. Chacur M. Biedenkapp J. O’Connor K. Poole S. (2003). Spinal glia and proinflammatory cytokines mediate mirror-image neuropathic pain in rats. *J. Neurosci.* 23 1026–1040. 12574433 10.1523/JNEUROSCI.23-03-01026.2003 PMC6741915

[B141] Mirsattari S. M. Power C. Nath A. (1999). Primary headaches in HIV-infected patients. *Headache* 39 3–10. 10.1046/j.1526-4610.1999.3901003.x 15613188

[B142] Moalem G. Tracey D. J. (2006). Immune and inflammatory mechanisms in neuropathic pain. *Brain Res. Rev.* 51 240–264. 10.1016/j.brainresrev.2005.11.004 16388853

[B143] Mocroft A. Ledergerber B. Katlama C. Kirk O. Reiss P. d’Arminio Monforte A. (2003). Decline in the AIDS and death rates in the EuroSIDA study: an observational study. *Lancet* 362 22–29. 10.1016/S0140-6736(03)13802-0 12853195

[B144] Moore K. A. Kohno T. Karchewski L. A. Scholz J. Baba H. Woolf C. J. (2002). Partial peripheral nerve injury promotes a selective loss of GABAergic inhibition in the superficial dorsal horn of the spinal cord. *J. Neurosci.* 22 6724–6731. 12151551 10.1523/JNEUROSCI.22-15-06724.2002 PMC6758148

[B145] Nath A. (2002). Human immunodeficiency virus (HIV) proteins in neuropathogenesis of HIV dementia. *J. Infect. Dis.* 186(Suppl. 2) S193–S198. 10.1086/344528 12424697

[B146] Nath A. Conant K. Chen P. Scott C. Major E. O. (1999). Transient exposure to HIV-1 Tat protein results in cytokine production in macrophages and astrocytes. A hit and run phenomenon. *J. Biol. Chem.* 274 17098–17102. 10.1074/jbc.274.24.17098 10358063

[B147] Ogoshi F. Yin H. Z. Kuppumbatti Y. Song B. Amindari S. Weiss J. H. (2005). Tumor necrosis-factor-alpha (TNF-alpha) induces rapid insertion of Ca2(+-permeable alpha-amino-3-hydroxyl-5-methyl-4-isoxazole-propionate (AMPA)/kainate (Ca-A/K) channels in a subset of hippocampal pyramidal neurons. *Exp. Neurol.* 193 384–393. 10.1016/j.expneurol.2004.12.026 15869941

[B148] Oh S. B. Tran P. B. Gillard S. E. Hurley R. W. Hammond D. L. Miller R. J. (2001). Chemokines and glycoprotein120 produce pain hypersensitivity by directly exciting primary nociceptive neurons. *J. Neurosci.* 21 5027–5035. 11438578 10.1523/JNEUROSCI.21-14-05027.2001 PMC6762869

[B149] Ohtori S. Takahashi K. Moriya H. Myers R. R. (2004). TNF-alpha and TNF-alpha receptor type 1 upregulation in glia and neurons after peripheral nerve injury: studies in murine DRG and spinal cord. *Spine (Phila Pa 1976)* 29 1082–1088. 10.1097/00007632-200405150-00006 15131433

[B150] Oka T. Oka K. Hosoi M. Hori T. (1995). Intracerebroventricular injection of interleukin-6 induces thermal hyperalgesia in rats. *Brain Res.* 692 123–128. 10.1016/0006-8993(95)00691-I 8548295

[B151] Okada-Ogawa A. Suzuki I. Sessle B. J. Chiang C. Y. Salter M. W. Dostrovsky J. O. (2009). Astroglia in medullary dorsal horn (trigeminal spinal subnucleus caudalis) are involved in trigeminal neuropathic pain mechanisms. *J. Neurosci.* 29 11161–11171. 10.1523/JNEUROSCI.3365-09.2009 19741123 PMC2804401

[B152] Onen N. F. Barrette E. P. Shacham E. Taniguchi T. Donovan M. Overton E. T. (2012). A review of opioid prescribing practices and associations with repeat opioid prescriptions in a contemporary outpatient HIV clinic. *Pain Pract.* 12 440–448. 10.1111/j.1533-2500.2011.00520.x 22103269 PMC3943214

[B153] Ozawa T. Nakagawa T. Shige K. Minami M. Satoh M. (2001). Changes in the expression of glial glutamate transporters in the rat brain accompanied with morphine dependence and naloxone-precipitated withdrawal. *Brain Res.* 905 254–258. 10.1016/S0006-8993(01)02536-7 11423104

[B154] Patel J. P. Sengupta R. Bardi G. Khan M. Z. Mullen-Przeworski A. Meucci O. (2006). Modulation of neuronal CXCR4 by the micro-opioid agonist DAMGO. *J. Neurovirol.* 12 492–500. 10.1080/13550280601064798 17162664 PMC2676683

[B155] Perl A. Banki K. (2000). Genetic and metabolic control of the mitochondrial transmembrane potential and reactive oxygen intermediate production in HIV disease. *Antioxid. Redox Signal.* 2 551–573. 10.1089/15230860050192323 11229368

[B156] Persidsky Y. Gendelman H. E. (2003). Mononuclear phagocyte immunity and the neuropathogenesis of HIV-1 infection. *J. Leukoc. Biol.* 74 691–701. 10.1189/jlb.0503205 14595004

[B157] Phillis J. W. Ren J. O’Regan M. H. (2000). Transporter reversal as a mechanism of glutamate release from the ischemic rat cerebral cortex: studies with DL-threo-beta-benzyloxyaspartate. *Brain Res.* 880 224 10.1016/S0006-8993(00)02755-4 11033012

[B158] Pocock J. M. Kettenmann H. (2007). Neurotransmitter receptors on microglia. *Trends Neurosci.* 30 527–535. 10.1016/j.tins.2007.07.007 17904651

[B159] Podhaizer E. M. Zou S. Fitting S. Samano K. L. El-Hage N. Knapp P. E. (2012). Morphine and gp120 toxic interactions in striatal neurons are dependent on HIV-1 strain. *J. Neuroimmune Pharmacol.* 7 877–891. 10.1007/s11481-011-9326-z 22101471 PMC3314128

[B160] Poole S. Cunha F. Q. Selkirk S. Lorenzetti B. B. Ferreira S. H. (1995). Cytokine-mediated inflammatory hyperalgesia limited by interleukin-10. *Br. J. Pharmacol.* 115 684–688. 10.1111/j.1476-5381.1995.tb14987.x 7582491 PMC1908480

[B161] Price D. D. (2000). Psychological and neural mechanisms of the affective dimension of pain. *Science* 288 1769–1772. 10.1126/science.288.5472.1769 10846154

[B162] Price D. D. Long S. Wilsey B. Rafii A. (1998). Analysis of peak magnitude and duration of analgesia produced by local anesthetics injected into sympathetic ganglia of complex regional pain syndrome patients. *Clin. J. Pain* 14 216–226. 10.1097/00002508-199809000-00008 9758071

[B163] Price M. P. McIlwrath S. L. Xie J. Cheng C. Qiao J. Tarr D. E. (2001). The DRASIC cation channel contributes to the detection of cutaneous touch and acid stimuli in mice. *Neuron* 32 1071–1083. 10.1016/S0896-6273(01)00547-5 11754838

[B164] Qin L. Block M. L. Liu Y. Bienstock R. J. Pei Z. Zhang W. (2005). Microglial NADPH oxidase is a novel target for femtomolar neuroprotection against oxidative stress. *FASEB J.* 19 550–557. 10.1096/fj.04-2857com 15791005

[B165] Raghavendra V. Rutkowski M. D. DeLeo J. A. (2002). The role of spinal neuroimmune activation in morphine tolerance/hyperalgesia in neuropathic and sham-operated rats. *J. Neurosci.* 22 9980–9989. 12427855 10.1523/JNEUROSCI.22-22-09980.2002 PMC6757841

[B166] Raghavendra V. Tanga F. DeLeo J. A. (2003). Inhibition of microglial activation attenuates the development but not existing hypersensitivity in a rat model of neuropathy. *J. Pharmacol. Exp. Ther.* 306 624–630. 10.1124/jpet.103.052407 12734393

[B167] Rahim R. T. Meissler J. J. Zhang L. Adler M. W. Rogers T. J. Eisenstein T. K. (2003). Withdrawal from morphine in mice suppresses splenic macrophage function, cytokine production, and costimulatory molecules. *J. Neuroimmunol.* 144 16–27. 10.1016/S0165-5728(03)00273-X 14597094

[B168] Reeve A. J. Patel S. Fox A. Walker K. Urban L. (2000). Intrathecally administered endotoxin or cytokines produce allodynia, hyperalgesia and changes in spinal cord neuronal responses to nociceptive stimuli in the rat. *Eur. J. Pain* 4 247–257. 10.1053/eujp.2000.0177 10985868

[B169] Ren K. Dubner R. (2010). Interactions between the immune and nervous systems in pain. *Nat. Med.* 16 1267–1276. 10.1038/nm.2234 20948535 PMC3077564

[B170] Ribeiro R. A. Vale M. L. Ferreira S. H. Cunha F. Q. (2000). Analgesic effect of thalidomide on inflammatory pain. *Eur. J. Pharmacol.* 391 97–103. 10.1016/S0014-2999(99)00918-8 10720640

[B171] Rivera-Amill V. Silverstein P. S. Noel R. J. Kumar S. Kumar A. (2010). Morphine and rapid disease progression in nonhuman primate model of AIDS: inverse correlation between disease progression and virus evolution. *J. Neuroimmune Pharmacol.* 5 122–132. 10.1007/s11481-009-9184-0 20013315 PMC4095870

[B172] Rothstein J. D. Dykes-Hoberg M. Pardo C. A. Bristol L. A. Jin L. Kuncl R. W. (1996). Knockout of glutamate transporters reveals a major role for astroglial transport in excitotoxicity and clearance of glutamate. *Neuron* 16 675–686. 10.1016/S0896-6273(00)80086-0 8785064

[B173] Rothstein J. D. Martin L. Levey A. I. Dykes-Hoberg M. Jin L. Wu D. (1994). Localization of neuronal and glial glutamate transporters. *Neuron* 13 713–725. 10.1016/0896-6273(94)90038-8 7917301

[B174] Schafers M. Geis C. Svensson C. I. Luo Z. D. Sommer C. (2003). Selective increase of tumour necrosis factor-alpha in injured and spared myelinated primary afferents after chronic constrictive injury of rat sciatic nerve. *Eur. J. Neurosci.* 17 791–804. 10.1046/j.1460-9568.2003.02504.x 12603269

[B175] Schmeichel A. M. Schmelzer J. D. Low P. A. (2003). Oxidative injury and apoptosis of dorsal root ganglion neurons in chronic experimental diabetic neuropathy. *Diabetes Metab. Res. Rev.* 52 165–171. 10.2337/diabetes.52.1.165 12502508

[B176] Scholz J. Broom D. C. Youn D. H. Mills C. D. Kohno T. Suter M. R. (2005). Blocking caspase activity prevents transsynaptic neuronal apoptosis and the loss of inhibition in lamina II of the dorsal horn after peripheral nerve injury. *J. Neurosci.* 25 7317–7323. 10.1523/JNEUROSCI.1526-05.2005 16093381 PMC6725303

[B177] Seifert F. Maihofner C. (2011). Functional and structural imaging of pain-induced neuroplasticity. *Curr. Opin. Anaesthesiol.* 24 515–523. 10.1097/ACO.0b013e32834a1079 21822136

[B178] Sekiguchi M. Sekiguchi Y. Konno S. Kobayashi H. Homma Y. Kikuchi S. (2009). Comparison of neuropathic pain and neuronal apoptosis following nerve root or spinal nerve compression. *Eur. Spine J.* 18 1978–1985. 10.1007/s00586-009-1064-z 19543754 PMC2899442

[B179] Sengupta R. Burbassi S. Shimizu S. Cappello S. Vallee R. B. Rubin J. B. (2009). Morphine increases brain levels of ferritin heavy chain leading to inhibition of CXCR4-mediated survival signaling in neurons. *J. Neurosci.* 29 2534–2544. 10.1523/JNEUROSCI.5865-08.2009 19244528 PMC2664553

[B180] Silva C. Zhang K. Tsutsui S. Holden J. K. Gill M. J. Power C. (2003). Growth hormone prevents human immunodeficiency virus-induced neuronal p53 expression. *Ann. Neurol.* 54 605–614. 10.1002/ana.10729 14595650

[B181] Simpson D. M. (2002). Selected peripheral neuropathies associated with human immunodeficiency virus infection and antiretroviral therapy. *J. Neurovirol.* 8(Suppl. 2) 33–41. 10.1080/13550280290167939 12491149

[B182] Singh I. N. El-Hage N. Campbell M. E. Lutz S. E. Knapp P. E. Nath A. (2005). Differential involvement of p38 and JNK MAP kinases in HIV-1 Tat and gp120-induced apoptosis and neurite degeneration in striatal neurons. *Neuroscience* 135 781–790. 10.1016/j.neuroscience.2005.05.028 16111829 PMC4310730

[B183] Sinkovics J. G. (1991). Programmed cell death (apoptosis): its virological and immunological connections (a review). *Acta Microbiol. Hung.* 38 321–334. 1817429

[B184] Smith H. S. (2011). Treatment considerations in painful HIV-related neuropathy. *Pain Physician* 14 E505–E524. 22086103

[B185] Sommer C. Kress M. (2004). Recent findings on how proinflammatory cytokines cause pain: peripheral mechanisms in inflammatory and neuropathic hyperalgesia. *Neurosci. Lett.* 361 184–187. 10.1016/j.neulet.2003.12.007 15135924

[B186] Sommer C. Schafers M. Marziniak M. Toyka K. V. (2001). Etanercept reduces hyperalgesia in experimental painful neuropathy. *J. Peripher. Nerv. Syst.* 6 67–72. 10.1111/j.1529-8027.2001.01010.x 11446385

[B187] Song P. Zhao Z. Q. (2001). The involvement of glial cells in the development of morphine tolerance. *Neurosci. Res.* 39 281–286. 10.1016/S0168-0102(00)00226-1 11248367

[B188] Stellwagen D. Beattie E. C. Seo J. Y. Malenka R. C. (2005). Differential regulation of AMPA receptor and GABA receptor trafficking by tumor necrosis factor-alpha. *J. Neurosci.* 25 3219–3228. 10.1523/JNEUROSCI.4486-04.2005 15788779 PMC6725093

[B189] Stiene-Martin A. Knapp P. E. Martin K. Gurwell J. A. Ryan S. Thornton S. R. (2001). Opioid system diversity in developing neurons, astroglia, and oligodendroglia in the subventricular zone and striatum: impact on gliogenesis in vivo. *Glia* 36 78–88. 10.1002/glia.1097.abs 11571786 PMC4303466

[B190] Stiene-Martin A. Zhou R. Hauser K. F. (1998). Regional, developmental, and cell cycle-dependent differences in mu, delta, and kappa-opioid receptor expression among cultured mouse astrocytes. *Glia* 22 249–259. 10.1002/(SICI)1098-1136(199803)22:3<249::AID-GLIA4>3.3.CO;2-0 9482211 PMC4319791

[B191] Sun Y. Sahbaie P. Liang D. Li W. Clark J. D. (2014). Opioids enhance CXCL1 expression and function after incision in mice. *J. Pain* 15 856–866. 10.1016/j.jpain.2014.05.003 24887006 PMC4131856

[B192] Sung B. Lim G. Mao J. (2003). Altered expression and uptake activity of spinal glutamate transporters after nerve injury contribute to the pathogenesis of neuropathic pain in rats. *J. Neurosci.* 23 2899–2910. 12684477 10.1523/JNEUROSCI.23-07-02899.2003 PMC6742068

[B193] Sung C. S. Wen Z. H. Chang W. K. Ho S. T. Tsai S. K. Chang Y. C. (2004). Intrathecal interleukin-1beta administration induces thermal hyperalgesia by activating inducible nitric oxide synthase expression in the rat spinal cord. *Brain Res.* 1015 145–153. 10.1016/j.brainres.2004.04.068 15223378

[B194] Svensson C. I. Schafers M. Jones T. L. Powell H. Sorkin L. S. (2005). Spinal blockade of TNF blocks spinal nerve ligation-induced increases in spinal P-p38. *Neurosci. Lett.* 379 209–213. 10.1016/j.neulet.2004.12.064 15843065

[B195] Sweitzer S. Martin D. DeLeo J. A. (2001). Intrathecal interleukin-1 receptor antagonist in combination with soluble tumor necrosis factor receptor exhibits an anti-allodynic action in a rat model of neuropathic pain. *Neuroscience* 103 529–539. 10.1016/S0306-4522(00)00574-1 11246166

[B196] Tadano T. Namioka M. Nakagawasai O. Tan-No K. Matsushima K. Endo Y. (1999). Induction of nociceptive responses by intrathecal injection of interleukin-1 in mice. *Life Sci.* 65 255–261. 10.1016/S0024-3205(99)00244-1 10447210

[B197] Tawfik V. L. Lacroix-Fralish M. L. Bercury K. K. Nutile-McMenemy N. Harris B. T. Deleo J. A. (2006). Induction of astrocyte differentiation by propentofylline increases glutamate transporter expression in vitro: heterogeneity of the quiescent phenotype. *Glia* 54 193–203. 10.1002/glia.20365 16819765

[B198] Tawfik V. L. Regan M. R. Haenggeli C. Lacroix-Fralish M. L. Nutile-McMenemy N. Perez N. (2008). Propentofylline-induced astrocyte modulation leads to alterations in glial glutamate promoter activation following spinal nerve transection. *Neuroscience* 152 1086–1092. 10.1016/j.neuroscience.2008.01.065 18358622 PMC2423012

[B199] Tomassini N. Renaud F. Roy S. Loh H. H. (2004). Morphine inhibits Fc-mediated phagocytosis through mu and delta opioid receptors. *J. Neuroimmunol.* 147 131–133. 10.1016/j.jneuroim.2003.10.028 14741444

[B200] Tortorici V. Robbins C. S. Morgan M. M. (1999). Tolerance to the antinociceptive effect of morphine microinjections into the ventral but not lateral-dorsal periaqueductal gray of the rat. *Behav. Neurosci.* 113 833–839. 10.1037/0735-7044.113.4.833 10495091

[B201] Toth P. T. Ren D. Miller R. J. (2004). Regulation of CXCR4 receptor dimerization by the chemokine SDF-1alpha and the HIV-1 coat protein gp120: a fluorescence resonance energy transfer (FRET) study. *J. Pharmacol. Exp. Ther.* 310 8–17. 10.1124/jpet.103.064956 15014135

[B202] Treede R. D. Jensen T. S. Campbell J. N. Cruccu G. Dostrovsky J. O. Griffin J. W. (2008). Neuropathic pain: redefinition and a grading system for clinical and research purposes. *Neurology* 70 1630–1635. 10.1212/01.wnl.0000282763.29778.59 18003941

[B203] Tsuda M. Shigemoto-Mogami Y. Koizumi S. Mizokoshi A. Kohsaka S. Salter M. W. (2003). P2X4 receptors induced in spinal microglia gate tactile allodynia after nerve injury. *Nature* 424 778–783. 10.1038/nature01786 12917686

[B204] Turchan-Cholewo J. Dimayuga F. O. Ding Q. Keller J. N. Hauser K. F. Knapp P. E. (2008). Cell-specific actions of HIV-Tat and morphine on opioid receptor expression in glia. *J. Neurosci. Res.* 86 2100–2110. 10.1002/jnr.21653 18338799 PMC2760290

[B205] Turchan-Cholewo J. Dimayuga F. O. Gupta S. Keller J. N. Knapp P. E. Hauser K. F. (2009). Morphine and HIV-Tat increase microglial-free radical production and oxidative stress: possible role in cytokine regulation. *J. Neurochem.* 108 202–215. 10.1111/j.1471-4159.2008.05756.x 19054280 PMC2749479

[B206] Ushijima H. Nishio O. Klocking R. Perovic S. Muller W. E. (1995). Exposure to gp120 of HIV-1 induces an increased release of arachidonic acid in rat primary neuronal cell culture followed by NMDA receptor-mediated neurotoxicity. *Eur. J. Neurosci.* 7 1353–1359. 10.1111/j.1460-9568.1995.tb01126.x 7582109

[B207] Viviani B. Bartesaghi S. Gardoni F. Vezzani A. Behrens M. M. Bartfai T. (2003). Interleukin-1beta enhances NMDA receptor-mediated intracellular calcium increase through activation of the Src family of kinases. *J. Neurosci.* 23 8692–8700. 14507968 10.1523/JNEUROSCI.23-25-08692.2003 PMC6740426

[B208] Wallace V. C. Blackbeard J. Pheby T. Segerdahl A. R. Davies M. Hasnie F. (2007a). Pharmacological, behavioural and mechanistic analysis of HIV-1 gp120 induced painful neuropathy. *Pain* 133 47–63. 10.1016/j.pain.2007.02.015 17433546 PMC2706950

[B209] Wallace V. C. Blackbeard J. Segerdahl A. R. Hasnie F. Pheby T. McMahon S. B. (2007b). Characterization of rodent models of HIV-gp120 and anti-retroviral-associated neuropathic pain. *Brain* 130 2688–2702. 10.1093/brain/awm195 17761732 PMC2656646

[B210] Walters E. T. (2014). Neuroinflammatory contributions to pain after SCI: roles for central glial mechanisms and nociceptor-mediated host defense. *Exp. Neurol.* 258 48–61. 10.1016/j.expneurol.2014.02.001 25017887

[B211] Walz A. Peveri P. Aschauer H. Baggiolini M. (1987). Purification and amino acid sequencing of NAF, a novel neutrophil-activating factor produced by monocytes. *Biochem. Biophys. Res. Commun.* 149 755–761. 10.1016/0006-291X(87)90432-3 3322281

[B212] Wang J. Barke R. A. Charboneau R. Roy S. (2005). Morphine impairs host innate immune response and increases susceptibility to *Streptococcus pneumoniae* lung infection. *J. Immunol.* 174 426–434. 10.4049/jimmunol.174.1.426 15611267

[B213] Wang W. Wang W. Wang Y. Huang J. Wu S. Li Y. Q. (2008). Temporal changes of astrocyte activation and glutamate transporter-1 expression in the spinal cord after spinal nerve ligation-induced neuropathic pain. *Anat. Rec.* 291 513–518. 10.1002/ar.20673 18384122

[B214] Wang Z. Pekarskaya O. Bencheikh M. Chao W. Gelbard H. A. Ghorpade A. (2003). Reduced expression of glutamate transporter EAAT2 and impaired glutamate transport in human primary astrocytes exposed to HIV-1 or gp120. *Virology* 312 60–73. 10.1016/S0042-6822(03)00181-8 12890621

[B215] Watkins L. R. Hutchinson M. R. Johnston I. N. Maier S. F. (2005). Glia: novel counter-regulators of opioid analgesia. *Trends Neurosci.* 28 661–669. 10.1016/j.tins.2005.10.001 16246435

[B216] Watkins L. R. Hutchinson M. R. Rice K. C. Maier S. F. (2009). The “toll” of opioid-induced glial activation: improving the clinical efficacy of opioids by targeting glia. *Trends Pharmacol. Sci.* 30 581–591. 10.1016/j.tips.2009.08.002 19762094 PMC2783351

[B217] Watkins L. R. Martin D. Ulrich P. Tracey K. J. Maier S. F. (1997). Evidence for the involvement of spinal cord glia in subcutaneous formalin induced hyperalgesia in the rat. *Pain* 71 225–235. 10.1016/S0304-3959(97)03369-1 9231865

[B218] Watkins L. R. Milligan E. D. Maier S. F. (2001). Glial activation: a driving force for pathological pain. *Trends Neurosci.* 24 450–455. 10.1016/S0166-2236(00)01854-3 11476884

[B219] Wei F. Guo W. Zou S. Ren K. Dubner R. (2008). Supraspinal glial-neuronal interactions contribute to descending pain facilitation. *J. Neurosci.* 28 10482–10495. 10.1523/JNEUROSCI.3593-08.2008 18923025 PMC2660868

[B220] Weng H. R. Aravindan N. Cata J. P. Chen J. H. Shaw A. D. Dougherty P. M. (2005). Spinal glial glutamate transporters downregulate in rats with taxol-induced hyperalgesia. *Neurosci. Lett.* 386 18–22. 10.1016/j.neulet.2005.05.049 15975716

[B221] Weng H. R. Chen J. H. Cata J. P. (2006). Inhibition of glutamate uptake in the spinal cord induces hyperalgesia and increased responses of spinal dorsal horn neurons to peripheral afferent stimulation. *Neuroscience* 138 1351–1360. 10.1016/j.neuroscience.2005.11.061 16426766

[B222] Wetzel M. A. Steele A. D. Eisenstein T. K. Adler M. W. Henderson E. E. Rogers T. J. (2000). Mu-opioid induction of monocyte chemoattractant protein-1, RANTES, and IFN-gamma-inducible protein-10 expression in human peripheral blood mononuclear cells. *J. Immunol.* 165 6519–6524. 10.4049/jimmunol.165.11.6519 11086093

[B223] Weyerbacher A. R. Xu Q. Tamasdan C. Shin S. J. Inturrisi C. E. (2010). N-Methyl-D-aspartate receptor (NMDAR) independent maintenance of inflammatory pain. *Pain* 148 237–246. 10.1016/j.pain.2009.11.003 20005044 PMC2831745

[B224] White F. A. Feldman P. Miller R. J. (2009). Chemokine signaling and the management of neuropathic pain. *Mol. Interv.* 9 188–195. 10.1124/mi.9.4.7 19720751 PMC2861804

[B225] White F. A. Jung H. Miller R. J. (2007). Chemokines and the pathophysiology of neuropathic pain. *Proc. Natl. Acad. Sci. U.S.A.* 104 20151–20158. 10.1073/pnas.0709250104 18083844 PMC2154400

[B226] Wieseler-Frank J. Maier S. F. Watkins L. R. (2005). Immune-to-brain communication dynamically modulates pain: physiological and pathological consequences. *Brain Behav. Immun.* 19 104–111. 10.1016/j.bbi.2004.08.004 15664782

[B227] Wilson N. M. Jung H. Ripsch M. S. Miller R. J. White F. A. (2011). CXCR4 signaling mediates morphine-induced tactile hyperalgesia. *Brain Behav. Immun.* 25 565–573. 10.1016/j.bbi.2010.12.014 21193025 PMC3039030

[B228] Wood J. N. Boorman J. P. Okuse K. Baker M. D. (2004). Voltage-gated sodium channels and pain pathways. *J. Neurobiol.* 61 55–71. 10.1002/neu.20094 15362153

[B229] Woolf C. J. Mannion R. J. (1999). Neuropathic pain: aetiology, symptoms, mechanisms, and management. *Lancet* 353 1959–1964. 10.1016/S0140-6736(99)01307-0 10371588

[B230] World Health Organization (1996). *Cancer Pain Relief. With a Guide to Opioid Availability* 2nd Edn. Geneva: World Health Organization.

[B231] Xin W. J. Weng H. R. Dougherty P. M. (2009). Plasticity in expression of the glutamate transporters GLT-1 and GLAST in spinal dorsal horn glial cells following partial sciatic nerve ligation. *Mol. Pain* 5 15 10.1186/1744-8069-5-15 PMC2676254 19323820

[B232] Xu J. T. Xin W. J. Zang Y. Wu C. Y. Liu X. G. (2006). The role of tumor necrosis factor-alpha in the neuropathic pain induced by Lumbar 5 ventral root transection in rat. *Pain* 123 306–321. 10.1016/j.pain.2006.03.011 16675114

[B233] Yadav A. Collman R. G. (2009). CNS inflammation and macrophage/microglial biology associated with HIV-1 infection. *J. Neuroimmune Pharmacol.* 4 430–447. 10.1007/s11481-009-9174-2 19768553 PMC5935112

[B234] Yoshimura M. Jessell T. M. (1989). Primary afferent-evoked synaptic responses and slow potential generation in rat substantia gelatinosa neurons in vitro. *J. Neurophysiol.* 62 96–108. 2754484 10.1152/jn.1989.62.1.96

[B235] Yoshimura T. Matsushima K. Oppenheim J. J. Leonard E. J. (1987). Neutrophil chemotactic factor produced by lipopolysaccharide (LPS)-stimulated human blood mononuclear leukocytes: partial characterization and separation from interleukin 1 (IL 1). *J. Immunol.* 139 788–793. 3298433

[B236] Yuan S. B. Shi Y. Chen J. Zhou X. Li G. Gelman B. B. (2014). Gp120 in the pathogenesis of human immunodeficiency virus-associated pain. *Ann. Neurol.* 75 837–850. 10.1002/ana.24139 24633867 PMC4077969

[B237] Zhai Q. Luo Y. Zhang Y. Berman M. A. Dorf M. E. (2004). Low nuclear levels of nuclear factor-kappa B are essential for KC self-induction in astrocytes: requirements for shuttling and phosphorylation. *Glia* 48 327–336. 10.1002/glia.20087 15390109

[B238] Zhang R. X. Li A. Liu B. Wang L. Ren K. Zhang H. (2008). IL-1ra alleviates inflammatory hyperalgesia through preventing phosphorylation of NMDA receptor NR-1 subunit in rats. *Pain* 135 232–239. 10.1016/j.pain.2007.05.023 17689191 PMC2323207

[B239] Zhang R. X. Liu B. Wang L. Ren K. Qiao J. T. Berman B. M. (2005). Spinal glial activation in a new rat model of bone cancer pain produced by prostate cancer cell inoculation of the tibia. *Pain* 118 125–136. 10.1016/j.pain.2005.08.001 16154703

[B240] Zhao C. M. Guo R. X. Hu F. Meng J. L. Mo L. Q. Chen P. X. (2012). Spinal MCP-1 contributes to the development of morphine antinociceptive tolerance in rats. *Am. J. Med. Sci.* 344 473–479. 10.1097/MAJ.0b013e31826a82ce 23187120

[B241] Zhuo M. (2002). Glutamate receptors and persistent pain: targeting forebrain NR2B subunits. *Drug Discov. Today* 7 259–267. 10.1016/S1359-6446(01)02138-9 11839523

[B242] Zou S. Fitting S. Hahn Y. K. Welch S. P. El-Hage N. Hauser K. F. (2011). Morphine potentiates neurodegenerative effects of HIV-1 Tat through actions at mu-opioid receptor-expressing glia. *Brain* 134 3616–3631. 10.1093/brain/awr281 22102648 PMC3235561

